# Identification of excitatory-inhibitory links and network topology in large-scale neuronal assemblies from multi-electrode recordings

**DOI:** 10.1371/journal.pcbi.1006381

**Published:** 2018-08-27

**Authors:** Vito Paolo Pastore, Paolo Massobrio, Aleksandar Godjoski, Sergio Martinoia

**Affiliations:** 1 University of Genova, Dept. of Informatics, Bioengineering, Robotics and System Engineering, Genova, Italy; 2 3Brain gmbh, Wädenswil, Switzerland; 3 CNR—Institute of Biophysics, Genova, Italy; Ghent University, BELGIUM

## Abstract

Functional-effective connectivity and network topology are nowadays key issues for studying brain physiological functions and pathologies. Inferring neuronal connectivity from electrophysiological recordings presents open challenges and unsolved problems. In this work, we present a cross-correlation based method for reliably estimating not only excitatory but also inhibitory links, by analyzing multi-unit spike activity from large-scale neuronal networks. The method is validated by means of realistic simulations of large-scale neuronal populations. New results related to functional connectivity estimation and network topology identification obtained by experimental electrophysiological recordings from high-density and large-scale (i.e., 4096 electrodes) microtransducer arrays coupled to *in vitro* neural populations are presented. Specifically, we show that: (i) functional inhibitory connections are accurately identified in *in vitro* cortical networks, providing that a reasonable firing rate and recording length are achieved; (ii) small-world topology, with scale-free and rich-club features are reliably obtained, on condition that a minimum number of active recording sites are available. The method and procedure can be directly extended and applied to *in vivo* multi-units brain activity recordings.

## Author summary

The balance between excitation and inhibition is fundamental for proper brain functions and for this reason is precisely regulated in adult cortices. Impaired excitation/inhibition balance is often associated with several neurological disorders, such as epilepsy, autism and schizophrenia. However, estimating functional inhibitory connections is not an easy task and few methods are available to identify such connections from electrophysiological data. Here we present a cross-correlation based method to identify both excitatory and inhibitory functional connections in large-scale neuronal networks. The method is applicable to both in vitro and in vivo spike data recordings. Once a connectivity map (i.e. a graph) is obtained, we characterized the associated topology by means of classical graph theory metrics to unveil functional architecture. In this work, we analyze in vitro cortical networks probed by means of large-scale microelectrode arrays (i.e., 4096 sensors) and we derive network topologies from spike data. The functional organization found is called “small-world and scale-free” and is the same organization found in cortical in vivo brain regions by means of different experimental methods. We also show that to obtain reliable information about network architecture at least a network with a hundred of nodes-neurons is needed.

## Introduction

Understanding the relationships between structure and function, dynamics and connectivity of neuronal circuits are a challenge of the modern neurosciences, especially as the characterization of neuronal interaction in terms of functional and effective connectivity [1–3] is concerned. Functional connectivity is an observable phenomenon defined as statistical dependency between remote neurophysiological events; it is usually inferred on the basis of correlations among neuronal activity measurements, by means of different approaches ranging from basic cross-correlation[4] to model-based methods[1, 5]. Effective connectivity refers explicitly to the influence that a neuron or neural system exerts on another one, either at synaptic or population level; it can be inferred by perturbing the activity of a neuron, and then by measuring the other neurons activity changes.Structural or anatomical connectivity is related to the physical connections (i.e., synapses) among neurons [2]. In this paper, we refer to the more general framework of functional connectivity, even if, by using the proposed correlation-based method, directed graphs (i.e. causal relationships) can be derived (cf. Materials and Methods sect.).

The complexity of the nervous system and the difficulties of multi-site parallel recordings in *in vivo* experimental models, hampered the systematic study of emergent properties of complex networks. At the same time, the availability of validated methods able of reliably inferring functional connections down to synaptic level is still limited. To this end, we adopted a reductionist approach making use of *in vitro* experimental models coupled to Micro-Electrode Arrays (MEAs). In this context, large-scale neural networks developing *ex vivo* and chronically coupled to MEAs [6], represent a well-established experimental system for studying the neuronal dynamics at population level [7]. Despite their simplicity, they show recurrent synchronized periods of activity, as also observed *in vivo* during sleep or anesthesia, and even quiet wakefulness [8, 9]. These model systems represent a good trade-off between controllability-observability and similarity to the *in vivo* counterpart, allowing accessibility and manipulation from both chemical and electrical point of view. Recent advances in multichannel recording techniques have made possible to observe the activities of thousands of neurons simultaneously with the acquisition of massive amount of empirical data [10]. These methods are very attractive since they allow the detailed monitoring of the on-going electrophysiological spatio-temporal patterns of complex networks [11–14].

Reconstructing the detailed functional connectivity of a neuronal network from spikes data is not trivial, and it is still an open issue, due to the complexities introduced by neuron dynamics and high anatomical interconnectivity [15, 16]. Statistical analysis of spike trains was pioneered by Perkel [17] and followed by more than four decades of methodology development in this area [18]. Cross-correlation based methods remain the main statistics to evaluate interactions among the elements in a neuronal network, and produce a weighted assessment of the connections strength. Weak and non-significant connections may tend to obscure the relevant network topology made up of strong and significant links, and therefore they are often discarded by applying an absolute or a proportionally weighted threshold [19]. Correlation-based techniques include independent components analysis, synchrony measures [20], cross-correlation [21, 22], correlation coefficients [7, 23], partial-correlation [24]. Other widespread techniques to infer functional connectivity are based on Information Theory (IT) methods [10, 25, 26], Granger causality [27, 28] and dynamical causal modeling [1]. With few exceptions [29, 30], all the recently introduced and revisited methods concentrate on excitation, ignoring inhibition or admitting the failure in reliably identifying inhibitory links [26].

In this work, we focus attention on cross-correlation histogram (CCH) based methods. We present a new algorithm able to efficiently and accurately infer functional excitatory and inhibitory links; we validate the method on simulated neuronal networks; finally, we study connection properties in large-scale *ex vivo* neuronal networks showing how to directly and reliably derive the topological properties of such networks.

There are three different connectivity conditions that, theoretically, influence the temporal correlation between neurons: pairs of excitatory neurons, pairs of inhibitory neurons, and inhibitory-excitatory pairs [31]. The first term is the one usually estimated and from which we obtain the inferred functional excitatory network usually represented by a (directed) graph. The second term is negligible as inhibitory-inhibitory links are physiologically very sparse [32]. The last term, when it is exerted by a GABAergic interneuron to cortical excitatory neurons, acts by reducing the activity and decreasing the spontaneous fluctuations (i.e., feedforward inhibition). On the contrary, when it is exerted by cortical excitatory neurons to GABAergic interneurons, it acts by increasing the activity of such neurons that, in turn, form inhibitory synaptic contacts with the glutamatergic cortical cells (i.e., feed-back inhibition) [33]. In other studies [34–36],it was noticed the primary effect of inhibition is a trough in the cross-correlogram: to detect this interaction a background of postsynaptic spiking against which the inhibitory effect may be exercised (i.e., high and tonic firing rates) is needed [22]. From experimental works related to *in vivo* multi-unit recordings, it was shown the sensitivity to excitation is much higher than the sensitivity to inhibition [37] (due to the low firing rates of neurons).

Finally, it should be underlined the analysis of interactions in neuronal networks is a quite demanding computational process, and all the currently proposed methods for analyzing multiple spike trains rely on quantities that need to be computed through intensive calculations [38]. By using the *ad-hoc* developed CCH, we could derive functional connectivity maps (both for excitation and inhibition) and to reliably extract topological characteristics from multiple spike trains in large-scale networks (i.e., thousands of neurons) monitored by large-scale MEAs (i.e., thousands of micro-transducers).

## Results

### Revealing excitatory and inhibitory connections: New and optimized cross-correlogram based approach

Starting from the standard definition of the cross-correlation [22] (cf., Materials and Methods sect.), we adopted the normalization approach described in [21, 39] to obtain the “raw” Normalized Cross-Correlation Histogram (NCCH). We formalized our hypothesis that, the extraction of negative peaks (rather than troughs) obtained by a filtering operation on the NCCH and followed by distinct thresholding operations for excitatory and inhibitory connections allows to identify a significant percentage of inhibitory connections with a high-level accuracy at low computational cost. Theoretically, cross-correlation is able to detect both an increase and a decrease of the synchrony between spike trains related to putative interconnected neurons. However, in real experimental data, the cross-correlogram is very jagged making difficult the detection of small peaks and troughs, and, apart from specific conditions (i.e., high and tonic firing rate) [4], hindering the detection of inhibition. Our approach consists in a simple post processing of the cross-correlation histogram, thus obtaining what we called Filtered and Normalized Cross-Correlation Histogram (FNCCH, curly brackets in Eq (1)).

Stated a reference neuron *x* and a target neuron *y*, Eq (1) provides the mathematical definition of the absolute peak of the FNCCH.

$$FNCCH_{xy_{peak}}=C_{xy}\left(\tau \right)|={\arg\;\max}_{t}\left\{\left|C_{xy}\left(t\right)-\frac{1}{W}\sum_{v=-\frac{W}{2}}^{v=\frac{W}{2}}C_{xy}\left(v\right)\right|\right\}$$ (1)

where *W* is the time window where FNCCH is evaluated. The filtering procedure (cf. Materials and Methods sect.) consists in subtracting the mean value of the cross-correlogram (in the time window *W*) from the values of the normalized cross-correlogram *C*_*xy*_*(*ν*)*, ν *∈* [*-W/2*, *W/2*]. The subsequent peaks extraction operation is performed by considering the absolute values, and it allows to compute the highest peak. In this way, it is possible to distinguish between peaks and troughs by taking into account the original signs: a positive value refers to an excitatory link, and a negative value refers to an inhibitory one. Details about further refinements needed to avoid detection of false inhibitory connections can be found in the Supplementary Information (cf., Sect. S1). In the next sections, we show the validation of the method with the aid of large-scale *in silico* networks; then, we present the results, in terms of functional connectivity maps and network topology, obtained from the analysis of multi-electrode parallel recordings of *in vitro* neuronal populations. Such populations are coupled to both 60 channels MEAs (MEA-60) and high-density MEAs with 4096 micro-transducers (MEA-4k) (cf. Materials and Methods sect.).

### Validation of the FNCCH by means of *in silico* neural networks

**Fig 1:**
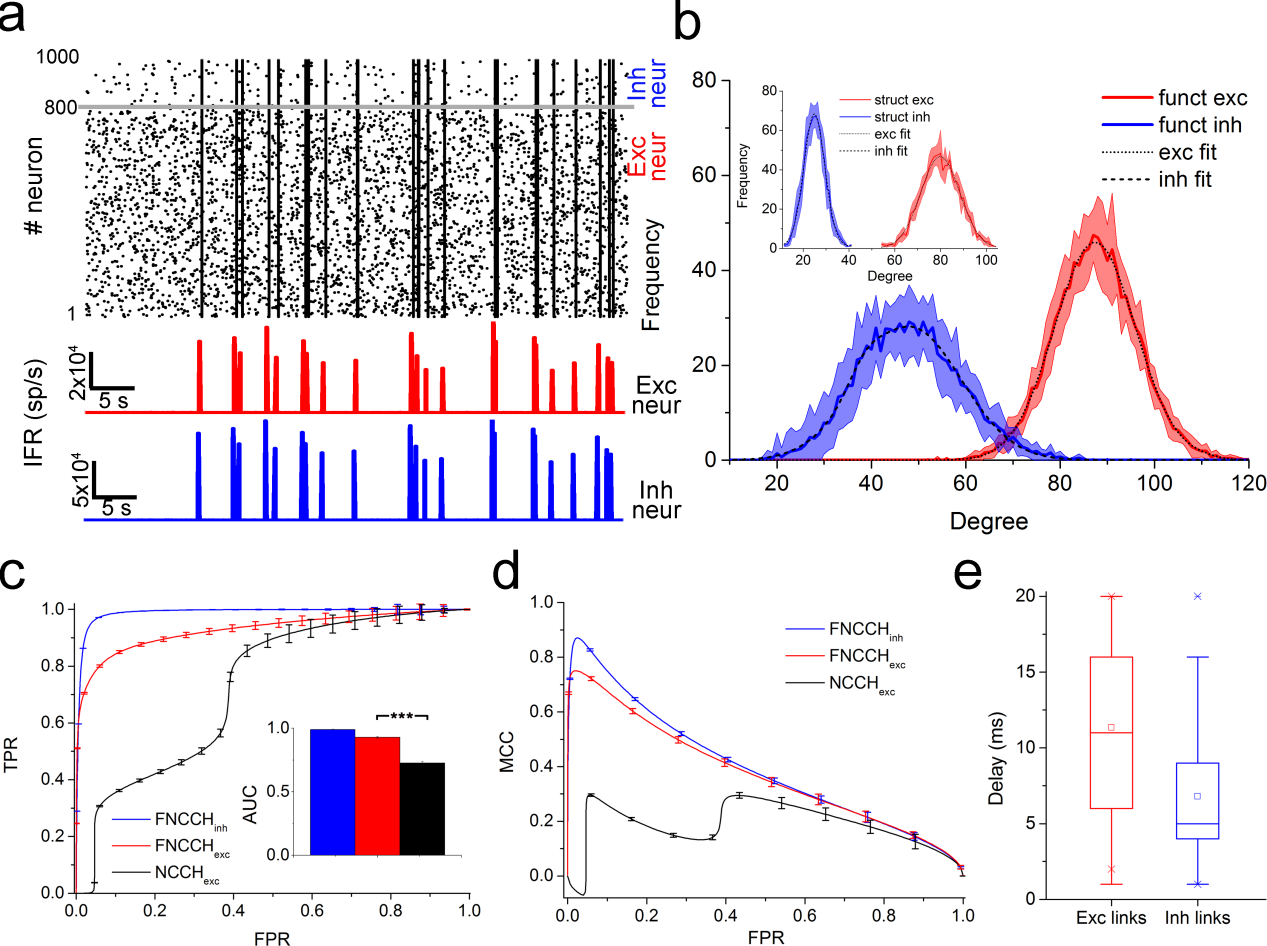
Functional connectivity estimation from 10 *in silico* neural networks. **a**, Raster Plot and mean Instantaneous Firing Rate (IFR) representative of the simulated electrophysiological activity. **b,** Estimated functional in-degree distribution (red curve for excitatory links and blue curve for the inhibitory ones) and (inset) structural in-degree distribution of the implemented network models. **c**, ROC functions for the inhibitory (blue curve) and the excitatory (red curve) links obtained by applying the FNCCH; the black curve, is related to only excitatory links extracted with the standard NCCH, is depicted for comparison. Corresponding AUCs are represented in the inset. **d**, MCC curves related to the inhibitory and excitatory links computed by applying the FNCCH; the black curve, related to only excitatory links extracted with the standard NCCH, is shown for comparison. **e**, Box plot of the excitatory and inhibitory delay distributions obtained by means of the FNCCH.

We applied the FNCCH (time window *W* = 25 ms and time bin 1.0 ms) to 10 realizations of *in silico* neural networks made up of 1000 randomly connected neurons, characterized by an average ratio between inhibitory and excitatory connections of 1/4 (cf., Materials and Methods sect.). The model was tuned to reproduce the dynamics exhibited by *in vitro* neuronal networks. Simulations show the typical signature characterized by a mix of spiking and bursting activities as displayed by the raster plot and the Instantaneous Firing Rate (IFR) traces of the excitatory (red) and inhibitory (blue) neuronal populations of Fig 1A. From a topological point of view, both the excitatory and inhibitory structural sub-networks follow a random connectivity, as the incoming degree distributions of Fig 1B (inset) display. Each neuron receives 100 connections from the other neurons: excitatory neurons receive 80% of excitatory and 20% of inhibitory links, respectively, (reflecting the ratio of the excitatory and inhibitory populations); inhibitory neurons receive only excitatory connections (cf. S2C Fig). Further details about the dynamics and connectivity of the simulated neuronal networks can be found in the Supplementary Information (cf., Sect. S2). Fig 1C and 1D quantify the performances of the FNCCH by means of the Receiver-Operating-Characteristic (ROC) [40] curve and the Matthews Correlation Coefficient (MCC) [41]. Fig 1C shows the ROC curves obtained by comparing the Synaptic Weight Matrix (SWM) of the model (i.e., the ground truth) with the computed Functional Connectivity Matrix (FCM), and Fig 1D shows the MCC curve (cf., Materials and Methods sect.). The ROC curve relative to the detection of inhibitory connections (blue curve in Fig 1C) is very close to the perfect classifier, with an Area Under Curve (AUC) of 0.98 ± 0.01 (blue bar in the inset of Fig 1C). The MCC curve relative to the inhibitory links (blue curve in Fig 1D) has a maximum value of 0.87 ± 0.04, showing a good precision in the identification of inhibition. Then, we compared the sensitivity of the FNCCH for the detection of excitatory links (red curves in Fig 1C and 1D) with the standard NCCH’s one (for excitation, black curves in Fig 1C and 1D) to underline the improved detection capabilities obtained by the filtering procedure. We observed not only a significant (p < 0.001) AUC increase (0.92 ± 0.01 vs. 0.72 ± 0.02, Fig 1C inset), but also significant improvements in both ROC and MCC curve shapes for low values of false positive rates (FPR). In particular, we can notice (Fig 1D), that the FNCCH excitatory curve has a maximum value of about 0.75 with respect to the correspondent NCCH value (for the same false positive rate) that is negative (suggesting a disagreement between prediction and observation). Further details about false and true positive detection can be found in the Supplementary Information (Sect. S5). The above results justify the use of a hard threshold procedure (cf., Materials and Methods sect.) to select the strongest and significant functional connections. The Thresholded Connectivity Matrix (TCM) is thus directly computed from the FCM by using a threshold equal to (*μ* + 1 *σ*), (mean plus one standard deviation of the connections strength) for the inhibitory links, and (*μ* + 2 *σ*) for the excitatory ones, obtaining estimated links with a very high-level of accuracy (cf. Materials and Methods sect.): R^2^ = 0.99 for the inhibitory links and R^2^ = 0.94 for the excitatory ones. To investigate whether the reconstructed functional connectivity network resembles the one of the model, we calculated the excitatory and the inhibitory (Fig 1B) links degree distribution after the thresholding procedure from TCM. The computed degree-distributions fit a Gaussian distribution (Fig 1B, R^2^ = 0.99 for the inhibitory links and R^2^ = 0.98 for the excitatory ones), in accordance with the original distributions used to generate the structural (random) connectivity of the model (Fig 1B inset). It can be noticed that the mean and standard deviation values of the functional Gaussian distribution for the excitatory links are in good agreement with the structural ones (*μ*_*funct*_ = 87, *σ*_*funct*_ = 13.2 and *μ*_*struct*_ = 80, *σ*_*struct*_ = 19.6). On the other hand, for the inhibitory links, such values are higher than the structural ones due to the presence of many polysynaptic interactions (*μ*_*funct*_ = 48, *σ*_*funct*_ = 9.3 and *μ*_*struct*_ = 25, *σ*_*struct*_ = 14.5). Finally, we computed the delay distribution for both the excitatory and the inhibitory links from the TCM (Fig 1E). The extracted delay distribution for the excitatory links qualitatively reflects the one used to generate the model (uniform distribution in the interval [0, 20] ms). The estimated inhibitory distribution, instead, exhibits a more confined range which reflects the one used to produce the model (constant delay set at 1 ms), but with a spread and a median value at about 5 ms (cf., Materials and Methods sect.). The disagreement can be explained by the presence of multiple and polysynaptic interactions (due to the combination of excitatory and inhibitory inputs on a single neuron; cf., Discussion sect.).

Further validation of the proposed method was pursued by implementing a scale-free (with small-world features) network. The results (cf. Supplementary Information, S3 Fig) are less striking than those obtained for random connectivity; nevertheless, FNCCH outperforms standard cross-correlation and the identification of inhibitory links is still maintained with a similar general trend.

### Functional Connectivity and emergent network topologies in *in vitro* large-scale neural networks

**Fig 2:**
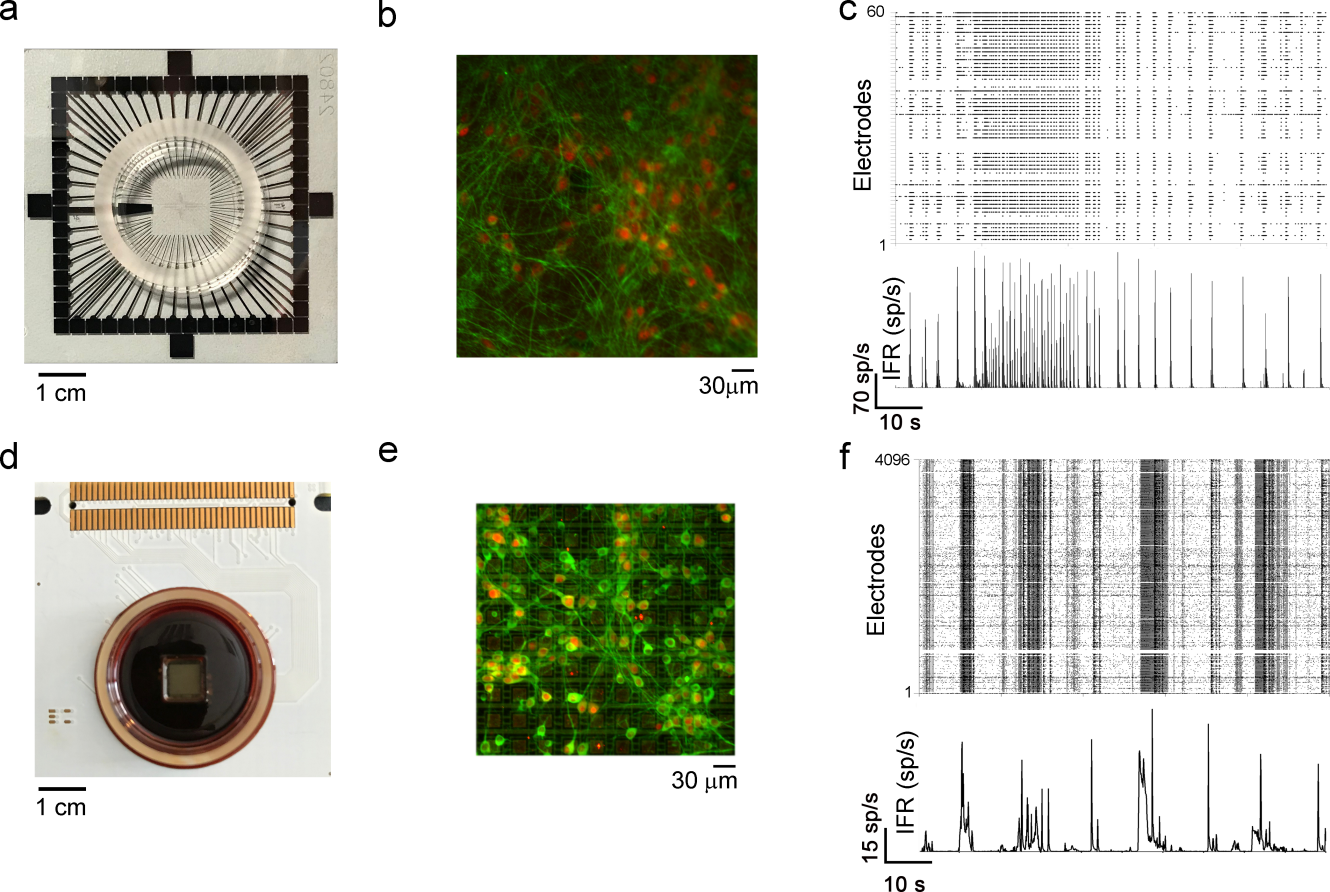
Micro-Electrode Arrays (MEAs) used in the experiments. **a,** MEA-60 device, **b**, Cortical network coupled to the MEA-60. **c**, example of 100 s recording of spontaneous electrophysiological activity and mean Instantaneous Firing Rate (IFR) plot. **d,** MEA-4k device. **e**, Cortical network coupled to the MEA-4k. **f**, Example of 100 s recording of spontaneous electrophysiological activity and mean IFR plot. Both the recordings come from cortical assemblies at DIV 25.

The FNCCH was applied to neuronal networks coupled to two different devices: MEA-60 and MEA-4k. Fig 2 shows the two utilized microtransducers (Fig 2A and 2D) and illustrative images of networks coupled to the two (Fig 2B and 2E). Such networks are the morphological substrate originating the complex electrophysiological activity characterized by an extensive bursting dynamics (i.e., highly synchronized network bursts) and a random spiking activity. Fig 2C and 2F show two examples of spontaneous activities recorded by a MEA-60 (Fig 2C) and a MEA-4k (Fig 2F). We can observe silent periods, desynchronized spiking activity, and peaks of activity (of different duration and called network bursts), which cause a rapid increase of the Instantaneous Firing Rate (IFR) (Fig 2C and 2F, bottom panels). More details about the spiking and bursting dynamics originated by networks coupled to MEA-4k are reported in the Supplementary Information (S1 Table). We analyzed three cortical and three striatal networks coupled to the MEA-60 (FNCCH parameters: time window *W* = 25 ms and time bin 0.1 ms) and three cortical networks coupled to the MEA-4k (FNCCH parameters: time windows *W* = 24 ms and time bin of 0.12 ms) after they reached a stable stage (i.e., after 21 Days *In Vitro*, 21 DIV).

**Fig 3:**
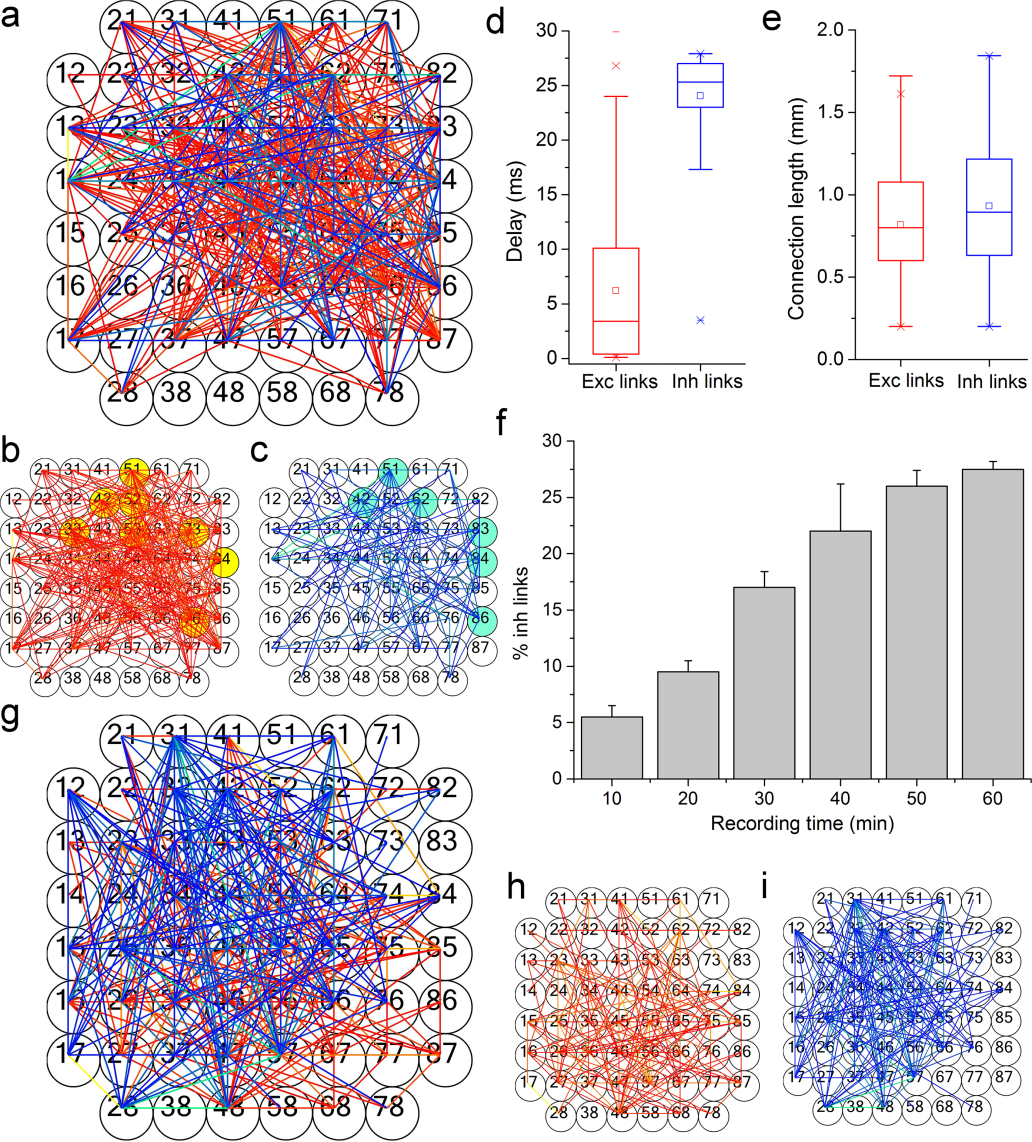
Functional connectivity analysis on different neural network populations coupled to the MEA-60 device. **a**, Functional connectivity graph obtained by applying the FNCCH to a cortical network at DIV 25. Excitatory and inhibitory links are separately thresholded and shown, for reader convenience, in panel **b,** (excitation, red color map) and **c,** (inhibition, blue color map). Color scales are indicative of the relative connection strength based on the peak of FNCCH. Yellow circles in panel **b** and cyan circles in panel **c** represent the identified rich club nodes. **d,** Box plot of the delays of the detected functional links. **e,** Box plot of the connection lengths of the detected links. **f,** Mean percentage of the inhibitory links revealed by the FNCCH at varying the recording time. **g**, Example of functional connectivity graph relative to a striatal network at DIV 21 coupled to a MEA-60 device. Panels **h,** and **i,** show the excitatory (red color map) and inhibitory (blue color map) networks, respectively.

**Fig 4:**
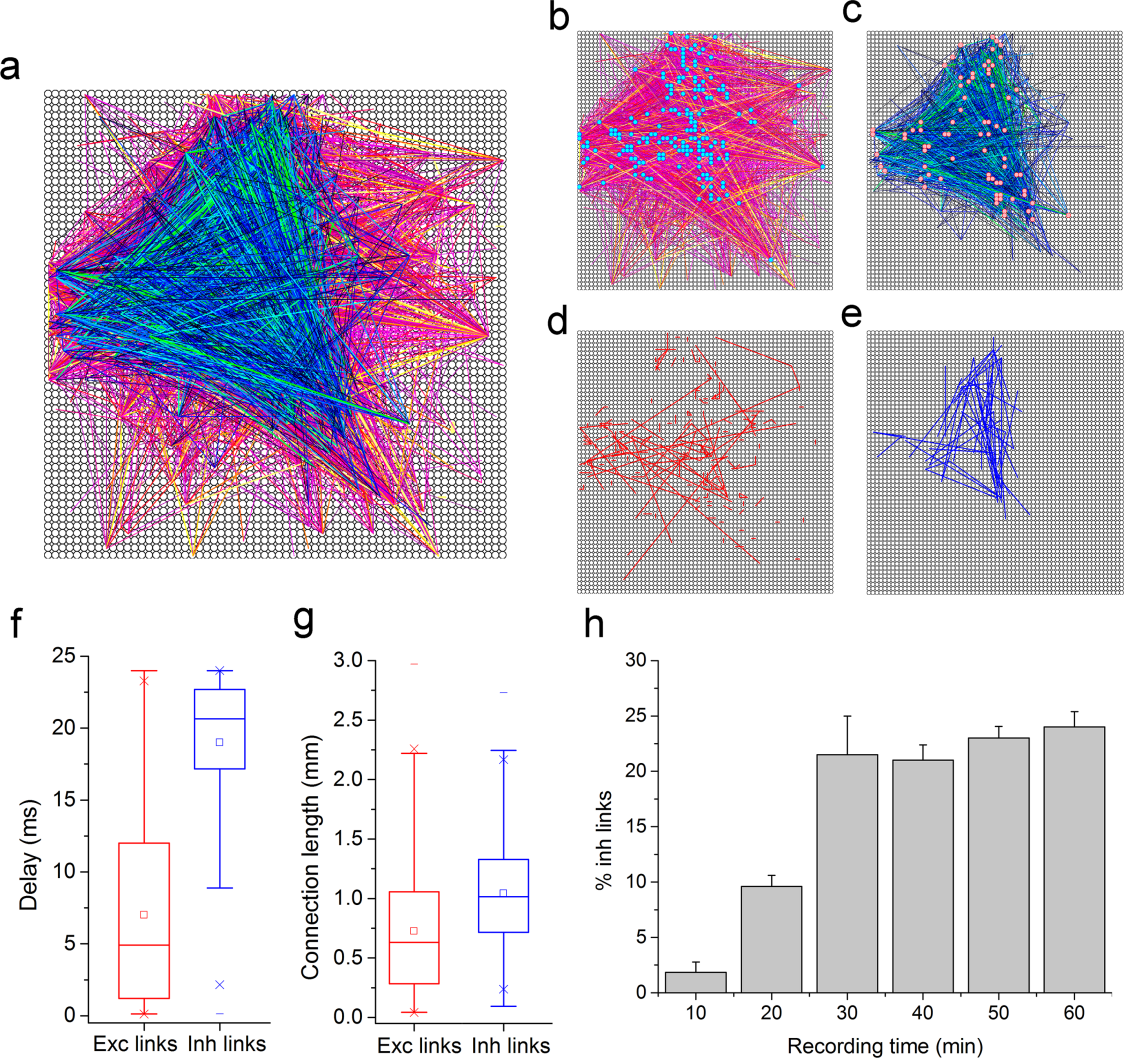
Functional connectivity analysis on cortex neural networks populations coupled to the MEA-4k. **a**, Functional connectivity graph obtained by applying the FNCCH to a cortical network at DIV 21 coupled to a MEA-4k device. Excitatory and inhibitory links are separately thresholded and shown, for reader convenience, in panel **b,** (excitation, red color map) and **c,** (inhibition, blue color map). Color scales are indicative of the relative connection strength based on the peak of FNCCH. Cyan circles in panel **b** and pink circles in panel **c** represent the identified rich club sub-networks. Functional connectivity maps showing the **d.** 200 excitatory and **e.** 50 inhibitory strongest links. **f,** Box plot of statistical distribution of the delays of the detected functional links. **g,** Box plot of the statistical distribution of the connection lengths of the detected links. **h,** Percentage of the inhibitory links revealed by the FNCCH at varying the recording time.

Fig 3A and 3G show connectivity graphs of cortical and striatal networks coupled to a MEA-60 device (Fig 3B and 3H and 3C and 3I show the contribution of excitation and inhibition, respectively). All the graphs were obtained by applying the hard threshold approach and the spatio-temporal filtering to prune co-activations (cf., Materials and Methods sect.). Then, we looked, for the cortical networks, the presence of privileged sub-networks constituted by the most connected nodes (i.e., rich club), by computing the Rich Club Coefficient (RCC) curve [42] (cf., Materials and Methods sect., Eq (10)). The nodes of these sub networks are highlighted in yellow and cyan (Fig 3B and 3C). For the striatal culture, the qualitative prevalence of inhibitory connections is clearly visible. To characterize the detected links for the cortical cultures, we computed the box plots of the functional connection peak delays (Fig 3D) and lengths (Fig 3E) of the excitatory (red) and inhibitory (blue) connections. Similar graphs derived from a cortical network coupled to a MEA-4k were obtained (Fig 4A). Links strength is represented by two color codes (arbitrary unit) for excitation (hot-red color code) and inhibition (cold-blue color code). The two detected subnetworks are also shown in Fig 4B and 4C. Moreover, the box plots pointing out the connection peak delays and lengths are depicted in Fig 4F and 4G. Noteworthy it is that the inhibitory links are slower, and with possible slightly longer connections than the excitatory ones, as reported in literature for structural and functional connectivity in brain slices [43]. Considering the high number of connections found by using the MEA-4k, we point out the two hundred strongest connections for excitation and the fifty strongest connections for inhibition (Fig 4D and 4E), illustrating how these main links include both short and long interactions with a prevalence of short interactions for excitatory connections.

We also computed the inhibitory links percentage with respect to the total number of detected links for the three different experimental conditions and three experiments for each condition. As expected, we found that striatal cultures have a higher percentage of inhibition and inhibitory links (about 60%)[44, 45] than cortical ones (about 25%). It is worth noticing that for the cortical cultures the excitatory/inhibitory ratio is detected quite independently of the number of recording sites (Figs 3F and 4H), although it tends to stabilize with a shorter recording time for the MEA-4k. Interestingly enough, the found ratio (about 1/4) in cortical networks between inhibitory and excitatory links is roughly the same as the ratio of inhibitory and excitatory neurons as estimated by immunostaining in similar experimental preparations [8].

**Fig 5:**
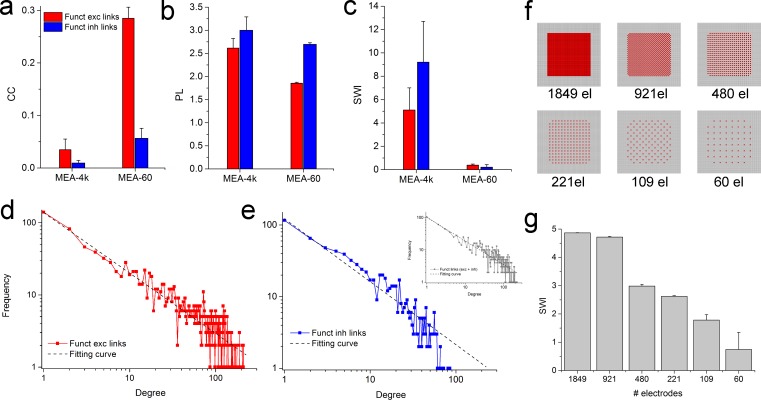
Topological features of the detected functional networks. **a**, Mean Cluster Coefficient (CC). **b,** average shortest Path Length (PL). **c**, Small-World Index (SWI). Red and blue colors indicate excitatory and inhibitory population, respectively. Degree Distributions of **d** excitatory, **e** inhibitory, and total links (inset). **f,** Schematic representation of the procedure used to decrease the electrodes density to analyze the SWI dependence on the electrodes resolution. **g,** SWI evaluation as a function of the electrodes density from 1849 to 60 microelectrodes.

In order to derive the topological features [46] of the analyzed cortical networks, we computed the Clustering Coefficient, CC (Fig 5A) and the average shortest Path Length, PL (Fig 5B). Then, we extracted the Small-World Index (SWI) by comparing the CC and the PL of the analyzed networks with the mean values of CC and PL of 100 realizations of a random network with the same degree-distribution, as recently proposed [26]. We found that when cortical networks are coupled to MEA-4ks devices, we can see the emergence of a clear small-world (SW) topology (Fig 5C); on the other hand, for cortical networks coupled to MEA-60s devices, we cannot infer any SW topology. From the measurements performed by MEA-4ks, we can state that both inhibitory and excitatory subnetworks with their small world index, SWI >>1 (9.2 ± 3.5 for the inhibitory links and 5.2 ± 2 for the excitatory ones) contribute to ‘segregation’. Moreover, both inhibitory and excitatory links with their fraction of long connections contribute also to network ‘integration’ (i.e., communication among the SWs). To further characterize the topology of these neuronal assemblies, we also investigated the possible emergence of scale-free topologies [47] by evaluating the presence of hubs[48] and power laws for the excitatory (Fig 5D), inhibitory (Fig 5E) and global (Fig 5E, inset) link degree distributions. In agreement with previously published model systems [49] and other studies [43], we obtained that such distributions fit a power law with R^2^ higher than 0.92, in all the three cases. Finally, we searched for the presence of privileged sub-networks made up of the most connected nodes (i.e., rich club) of the investigated networks by computing the RCC curve. For the analyzed cortical cultures, we found privileged sub-networks as indicated by the computed RCC values with a maximum value of 2.7 ± 0.5. Fig 4B and 4C show the rich club networks identified for one neural network coupled to the MEA-4k, represented by means of blue circles (for excitatory subnetwork) and pink circles (for inhibitory subnetworks). Fig 3B and 3C are the analogous for a cortical neural network coupled the MEA-60 (yellow for the excitatory nodes and light blue for the inhibitory ones).

Similar cortical networks coupled to the MEA-60 devices show no clear SW topology (Fig 5C); these networks seem to be characterized by a (sub)-random topology with SWI of 0.4 ± 0.1 for the excitatory and 0.2 ± 0.2 for the inhibitory links. These cortical networks are of the same type as the ones coupled to the MEA-4k (i.e., similar density of neurons, same age, same culture medium), and the apparent estimated random topology should be attributed to the low number of recording sites (i.e., 60 channels) that are not enough to reliably infer topological features. To determine how the number and density of electrodes are crucial, we computed the SWI by considering a reduced number of electrodes for the functional connectivity analysis from the MEA-4k recording, as described in Fig 5F. In particular, we started from the full resolution of the MEA-4k (i.e., 4096 electrodes), and we progressively decreased the electrode density to 60 electrodes (inter-electrode distance 189 μm, electrode density 19 electrode/mm^2^) to obtain a configuration comparable with the MEA-60 devices, as previously reported[50]. The obtained results are shown in Fig 5G: the SWI decreases down to a random topology becoming variable and unstable when the number of the considered electrodes is less than 100. This last result is referred to the excitatory links and the same analysis was not applied to the inhibitory connections. Such inhibitory links are much less than the excitatory ones, thus leading to an inhibitory topology reconstruction that is strongly influenced by the decimation scheme applied to reduce the number of electrodes.

## Discussion

The computation of the correlation of firing activity in the framework of multiple neural spike trains has been introduced since the 1960s. For over thirty years, cross-correlation, its generalizations [51], and its homologue in the frequency domain [52], have been the main tools to characterize interactions between neurons organized into functional groups, or “neuronal assemblies”. A common established technique was to build a cross-correlogram (CCH), describing the firing probability of a neuron as a function of time elapsed after a spike occurred in another one. Nevertheless, in the literature, there is no standard definition of CC, and the strength of a connection can be estimated by different means. To make the correlation coefficient independent of modulations in the firing rate, it is essential to have procedures for correction, normalization and thresholding of the coincidence counts obtained from cross-correlation calculations. Commonly used normalization procedures are related to Normalized Cross-Correlation Histogram (NCCH) [21, 39], event synchronization [53], Normalized Cross-Correlation (NCC–Pearson Coefficient) [23], Coincidence Index of the CC [26]. Once that a Functional Connectivity Matrix (FCM) is obtained, a thresholding procedure is necessary to discard those values that are related to spurious connections. All these approaches present advantages and disadvantages, but none of them have been applied to reliably identify inhibitory connections on large-scale network from spiking activity. In this paper we presented a filtered and normalized CC based algorithm (i.e., FNCCH) from which thresholded functional connectivity matrices and (directed) weighted graphs for excitation and inhibition can be obtained.

From the analysis of the data, we identified both small-world and scale free topologies in cortical networks for the excitatory and inhibitory sub-networks. More specifically, we extracted inhibitory networks in cortical (and striatal) neuronal cultures demonstrating the good performance of the method and offering new understanding of neuronal interactions in large cell populations. Finally, the proposed algorithm strengthens previously results presented in the literature [34], states a new way (i.e., through large-scale MEAs and CCH based analysis) to investigate network topology and opens up new perspective for the analysis of multisite electrophysiological recordings [54].

### Identification of inhibition

Generally, by inspecting a CCH, we can notice an increase or a decrease of the fluctuations [22]. In some studies, it was noticed that the primary effect of inhibition on the cross-correlogram is a trough near the origin, and for this interaction to be visible there must be present a background of postsynaptic spiking against which the inhibitory effect may be exercised (high-tonic firing rate regime) [4, 35]. From experimental works related to the analysis of connectivity from cortical multi-unit recordings [55], a good sensitivity for excitation is obtained, while the situation is considerably worse for inhibition.

This is due to a low sensitivity of CCH for inhibition, especially under the condition of low firing rates [4, 56]. The difference in sensitivity may amount to an order of magnitude, and it was demonstrated that for inhibition, the magnitude of the departure relative to the flat background is equal to the strength of the connection, whereas for excitation it involves an additional gain factor [4].

As a whole, the lack of efficiency in the detection of inhibition, simply reflects the disproportionate sensitivity of the analysis tool [57]. In our work, we introduced a cross-correlogram filtering approach (FNCCH) developed to overcome the inhibition detectability issue. As Fig 1 shows, the FNCCH is able to detect, with high accuracy, the inhibitory links when applied to *in silico* neural networks with similar dynamics with respect to the actual ones. The filtering procedure improves also the detectability of the excitatory links, resulting in a reshaping of the ROC curve (Fig 1A) with an increase of both precision (MCC curve, Fig 1B) and AUC with respect to the standard cross-correlation (NCCH). However, the presented FNCCH, being a CC-based method, has some limitations in the inhibitory links detection that we tried to investigate with our *in silico* models. The main factor affecting the detectability of inhibition, is the variability of CC. In order to reduce this variability, it is possible to increase the number of coincidences per bin by widening the bin-width (that is, down-sampling with loss of information in the acquired electrophysiological data), or by increasing the number of involved events (which can be obtained with high firing rate and/or by raising the recording time)[58]. Another influencing factor depends on the balance of excitatory and inhibitory neuronal inputs (i.e., balanced model) and it is referred to the relative strength between inhibitory and excitatory inputs. In fact, when the neuron is not balanced, excitation is, on average, stronger than inhibition. Conversely, when the neuron is balanced, both excitation and inhibition are strong and detection of inhibitory links improves [22, 31, 57]. Starting from the *in silico* model, we were able to investigate the impact of rates variability on excitation/inhibition detectability, and to try to define a reasonable threshold (criterion for detectability [22, 56]). In particular, we varied the firing rate of the inhibitory neurons from 20 spikes/s to 2 spikes/s, while maintaining a firing rate of 2–3 spikes/s for the excitatory neurons. We found that the detectability of the functional inhibitory links is preserved with our method, down to a firing of about 6 spikes/s, and then decreases significantly. We investigated also the inhibition identification with respect to the recording time. Starting from 1 hour of simulation, we reduced (10 min steps) the recording time, and we found that there is a decrease in the inhibition detectability below 30 minutes of recording (cf. S4 Fig). Finally, we investigated the performances of the FNCCH in a scale-free and small-world network. The detection of inhibition was still possible with relatively good results, even if the global performances of the algorithm decreases. This shall not be attributed to the scale-free topology, but to the reduced firing rate for both inhibitory and excitatory neurons and to possible unbalances between inhibition and excitation (cf. S3 Fig). Nevertheless, the method could reliably capture the topology of the network and qualitatively estimate the synaptic in-degree distribution. Thus, the obtained results enabled us to apply the FNCCH to *in vitro* large-scale neural networks, and allowed us to infer topology and functional organization. The described procedure could be also directly applied to Multi Unit Activity (MUA) from *in vivo* multi-site measurement recordings. Other methods (e.g., partial correlation, transfer entropy) were not taken explicitly into consideration for comparison, either for their computational costs, or for the inability to identify inhibitory links [59].

### The emergence of a scale free and small-world topology

The cortical networks probed with MEA-4k showed a clear small-world topology. The inhibitory functional links had a SWI equal to 9.2 ± 3.5, higher than the value extracted from the excitatory links (5.1 ± 1.9). Conversely, the cortical networks coupled to the MEA-60 showed a random organization topology (0.21 ± 0.212 for the inhibitory links and 0.38 ± 0.1 for the excitatory ones). These apparent random organizations are due to the low number of recording sites of the acquisition system; in fact, it is worth to remember that the SWI is computed by comparing cluster coefficient (CC) and average shortest path length (PL) of the analyzed networks to the corresponding values for surrogate random equivalent networks (same number of nodes and links). From the obtained results, unlike recently presented findings [42], we demonstrated that the emergence of small-worldness, cannot be reliably derived or observed in a neuronal population probed by a reduced number (< 100) of recording sites. To characterize connectivity properties, besides the importance of well-defined statistical tools used for the analysis, it is fundamental to probe network activity by using large-scale microtransducer arrays (i.e., with at least 200 electrodes). As a whole, the issue related to the low number of recording sites should be carefully taken into account when extracting dynamical features as well as organizational principles of complex networks.

Finally, it should be underlined that we focused our attention on CC based methods. We mentioned, in the Introduction, the widespread use of Information Theory (IT) based techniques. Beside the relative novelties of such methods, and the good performances (for a review see [38] and references therein), they showed high computational costs and, to our knowledge, the inability to reliably estimate inhibitory connections [26]. Although theoretically, IT based methods such as Transfer Entropy (TE) and Mutual Information (MI) are able to detect inhibitory links, we are not aware of studies consistently reporting a successful identification of inhibitory connections. The problem is in the incapability of distinguishing between excitatory and inhibitory links, rather than in the detection of inhibition as pointed out in the Supplementary Information (S6 Fig).

## Materials and methods

### Ethics statement

Primary neurons were obtained from rat embryos (18 days, E18) from Sprague Dawley pregnant rats (Charles River Laboratories). The experimental protocol was approved by the European Animal Care Legislation (2010/63/EU), by the Italian Ministry of Health in accordance with the D.L. 116/1992 and by the guidelines of the University of Genova (Prot. N. 24982, October 2013).

### Cross-correlation

Cross-correlation (CC) [22] measures the frequency at which a neuron or electrode fires (“target”) as a function of time, relative to the firing of an event in another one (“reference”). Mathematically, the correlation function is a statistic representing the average value of the product of two random processes (the spike trains). Given a reference electrode *x* and a target electrode *y*, the correlation function reduces to a simple probability *C*_*xy*_*(τ)* of observing a spike in one train *y* at time (*t + τ*), given that there was a spike in a second train *x* at time *t*; *τ* is called the time shift or the time lag. In this work, we use the standard definition for the cross-correlation computation, following a known normalization approach on the CC values [39]. We define the cross-correlation as follows:

$$C_{\mathrm{xy}}(\tau )=\frac{1}{\sqrt{N_{x}N_{y}}}\sum_{s=1}^{N_{x}}x(t_{s})y(t_{s}-\tau )$$ (2)

where *t*_*s*_ indicates the timing of a spike in the *x* train, *N*_*x*_ is the total number of spikes in the *x* train and *N*_*y*_ is the total number of spikes in the y train. Cross-correlation is limited to the interval [0, 1] and it is symmetric *C*_*xy*_(*τ*) *= C*_*yx*_(*-τ*). The cross-correlogram is then defined as the correlation function computed over a chosen correlation window (*W*, *τ =* [*-W/2*, *W/2*]). Different shapes of cross-correlograms can be obtained from pairs of analyzed spike trains. The occurrence of significant departures from a flat background in the cross-correlogram (i.e., a peak or a trough) is an indication of a functional connection[4]. In particular, a peak corresponds to an excitatory connection and a trough to an inhibitory link. The different amplitude of the peaks can be related to the existence of different levels of synchronization between neural spike trains. Generally, a correlogram can reflect a so-called direct excitatory connection between two neurons when a one-sided peak is evident (displaced from the origin of time by latency corresponding to the synaptic delay).

### Cross-correlation histogram

**Fig 6:**
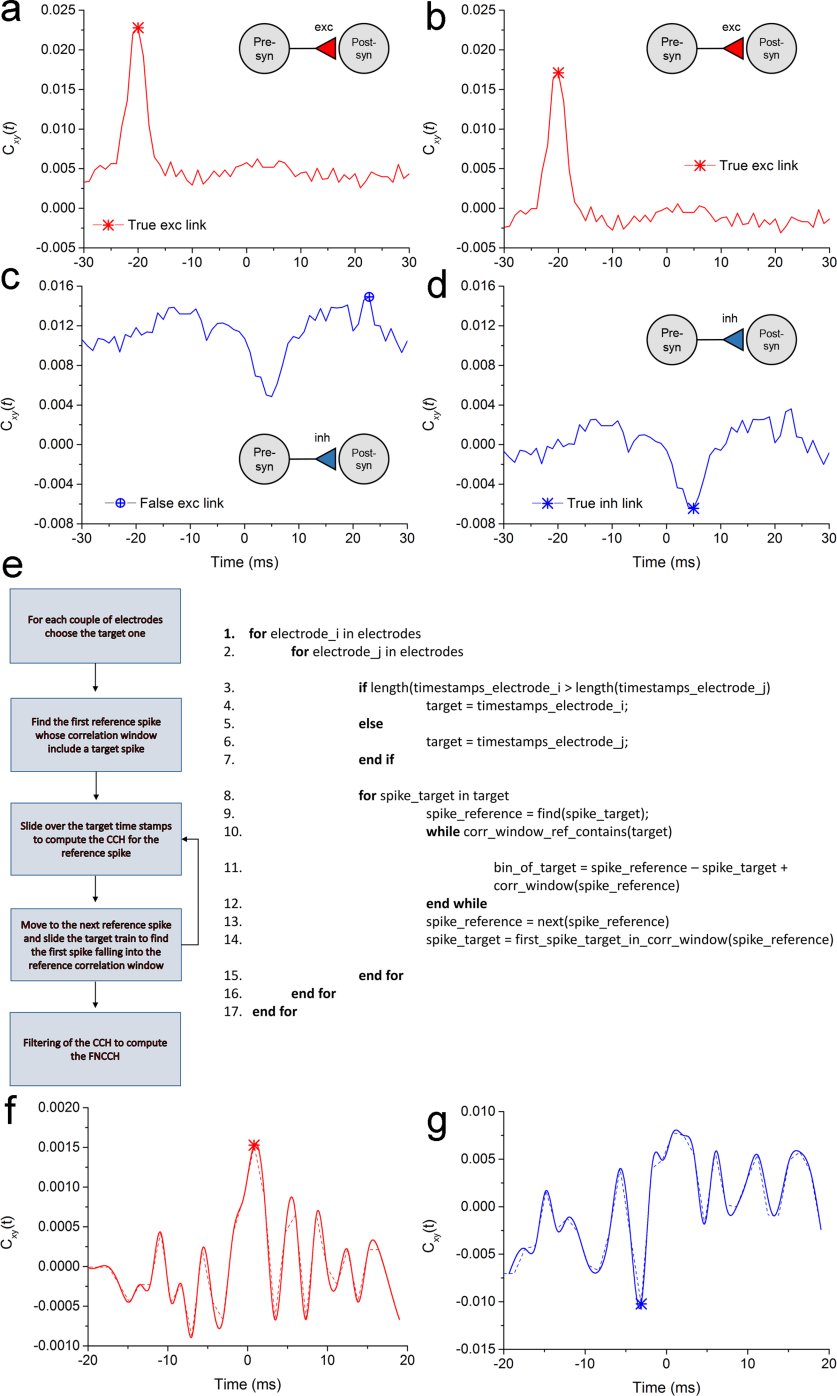
Example of detection for excitatory and inhibitory links in a network model. **a**, NCCH computed between two spike trains related to two neurons linked by an excitatory link in the model (identified by a red asterisk). **b**. FNCCH of the two neurons of panel **a**. The “entity peak” allows a better recognition of the excitatory link. **c**. NCCH computed between two spike trains related to two neurons linked by an inhibitory link in the model. The NCCH might detect a false excitatory peak (blue cross). **d**, FNCCH of the two neurons of panel **c**. The filtering procedure allows to recognize the through and to detect the negative peak correspondent to the inhibitory link (blue asterisk). **e.** Schematic representation and description of the algorithm to obtain the FNCCH. **f.** FNCCH (dashed line) for two putative neurons connected by a putative excitatory connection. **g.** FNCCH (dashed line) for two putative neurons connected by a putative inhibitory connection. The continuous lines in panels **f** and **g** are a smoothing of the histogram (dashed lines).

The use of spike train data offers the possibility to optimize the cross-correlation algorithm efficiency. To overcome the lack of efficiency of many of the proposed CC computation strategies, we present an alternative approach based on the “direct” spike time stamps inspection that avoids un-necessary calculations on the binarized spike trains. In fact, the only important information is stored in the bins containing a spike (i.e., spike time stamp), that are significantly less than null bins. If we consider that the average mean firing rate in neural networks oscillates between 0.2 spikes/s and 20 spikes/s [60], at a sampling frequency of 10 kHz, it yields only 2% of bin with spikes and 98% of meaningless bins: thus, we developed a new version of the CCH as indicated in Fig 6E.

### Filtered and normalized cross-correlation histogram (FNCCH)

Let us consider a reference neuron *x* and a target neuron *y*, and let us suppose that we computed the NCCH between *x* and *y*. After the NCCH computation, the maximum value (i.e., the peak) is used as a value reflecting the strength of the estimated functional link. If *x* and *y* share an excitatory link, this procedure works well (Fig 6A and 6B). On the other hand, when *x* inhibits *y*, the inhibitory trough will be discarded in favor of the NCCH peak (Fig 6C), with a misleading excitatory link detection. The CCH shapes are similar also in the correlograms derived from experimental data, although with an even more jagged behavior. Fig 6F and 6G show two examples of detected putative excitatory and inhibitory connections.

Eq (1) gives the mathematical definition of the FNCCH computation that overcomes this problem. We refer to the filtered peak value as *entity of the peak*. In this way, it is possible to distinguish between peaks and troughs by taking into account the sign. A positive peak is referred to an excitatory links (Fig 6A and 6B), conversely, a negative peak is referred to an inhibitory link (Fig 6D). We implemented and applied also a post-computation filtering procedure to improve the detectability of inhibitory links on noisy spike trains (cf., Supplementary Information, S1 Fig).

The block diagram and pseudocode depicted in Fig 6E show the sequence of operations executed by the FNCCH. The starting point is the first bin containing a spike in the target train. The binning procedure is directly performed on the time stamps. For each couple of neurons, starting from the first spike of the target train, we slide the time stamps of the reference electrode to find the first spike whose correlation window contains the target spike. Then, we continue to move over the target train to build the entire cross-correlogram (for that reference spike). When the correlation window for the reference spike is completed (i.e., when we have counted the number of spikes for all the bin of the target spike train), we move to the next spike of the reference train, and re-iterate the procedure starting from the first target spike into the correlation window, centered at the current reference spike. Then, we normalize the CC and repeat all the aforementioned operations for the other electrodes. Exploiting the symmetry of the CC function we consider only half of the electrodes for the computation. Moreover, for each pair, we select, as target train, the one with the smallest number of spikes to reduce the number of operations. Once the NCCH is obtained, we apply the filtering operation described by Eq (1) to compute the FNCCH values. Finally, we take the maximum absolute value as estimation of the correlation strength between the two electrodes. If it is negative, the found connection is considered a putative inhibitory link, otherwise is considered an excitatory one.

### Spatio-temporal filtering procedure

We applied a Spatio-Temporal Filter directly to the functional connectivity matrix (FCM) originated by the FNCCH. The procedure we implemented follows the one devised by Maccione et al.,[61] by defining a distance-dependent latency threshold. More in detail, we evaluated the links length (using the Euclidean distance) and the functional delays for each electrodes pair. We assumed as maximum propagation velocity a value of 400 mm/s[62]. If a functional connection has a temporal delay not compatible with such maximum velocity, it is discarded. Finally, we introduced also a minimum delay of about 1 ms, compatible with fast excitatory AMPA synaptic transmission.

Thus, we refined the FCM by removing all the links related to putative non-physiological connections.

### Thresholding procedure

Cross-correlation, as well as any other connectivity method, provides a full *n* x *n* Connectivity Matrix (CM), whose generic element (*i*, *j*) is the estimation of the strength of connection between electrodes *i* and *j*. A thresholding procedure is thus needed to eliminate those values that are only relative to noise and not to real functional connections. In the literature, there are several thresholding procedures, with different levels of complexity: the simplest one is to use a hard threshold, defined as (*μ* + *n* · *σ*), where *μ* and *σ* are the mean and the standard deviation computed among all the CM’s elements, respectively, and *n* is an integer[24]. There are other more sophisticated approaches based on shuffling methods that consist in destroying all the temporal correlations within the spike trains and compute a null hypothesis to test the significance of the connections[63]. However, shuffling procedures require many resources in terms of memory and computational times. In this work we proved, by means of the *in silico* network model, that a simple hard threshold method is sufficient. We found that significant levels of accuracy can be obtained with a threshold equal to (*μ* + *σ*) for both excitatory and inhibitory links (cf., Results sect.).

### Receiver operating characteristic (ROC) curve

The ROC curve[40] is a common metrics used to evaluate the performances of a binary classifier by comparing prediction and observation. In our study, the prediction is represented by the computed Thresholded functional Connectivity Matrix (TCM), and the observation corresponds to the Synaptic Weight Matrix (SWM) of the neural network model (i.e., the ground truth).

We can define the True Positive Rate (TPR) and the False Positive Rate (FPR) as follows:

$$TPR=\frac{TP}{TP+FN}$$ (3)

$$FPR=\frac{FP}{FP+TN}$$ (4)

where TP are the True Positive links, and TN, FP and FN are the True Negative, False Positive and False Negative connections, respectively. The ROC curve is then obtained by plotting TPR versus FPR. The Area Under Curve (AUC) is a main parameter extracted to have a single number describing the performances of a binary classifier: a random guess will correspond to 0.5, while a perfect classifier will have a value of 1. Another important metrics that can be extracted from a ROC analysis is the accuracy, defined as:

$$ACC=\frac{TP+TN}{TP+TN+FP+FN}$$ (5)

### Matthews correlation coefficient (MCC) curve

The MCC curve[40] is a common metrics, alternative to the ROC analysis, used to evaluate the performances of a binary classifier by comparing prediction and observation. Using the quantities defined in the previous paragraph, changing the threshold used to compute the TCM, we can define MCC as:

$$MCC=\frac{TP*TN-FP*FN}{\sqrt{\left(TP+FP)(TP+FN)(TN+FP)(TN+FN\right)}}$$ (6)

The MCC assumes values in the interval [–1, 1] and the MCC curve is obtained by plotting the MCC value versus the false positive rate.

### Cluster coefficient (CC)

Let *x* be a generic node and *v*_*x*_ the total number of nodes adjacent to *x* (including *x*). Let *u* be the total number of edges that actually exist between *x* and its neighbors. The maximum number of edges that can exist among all units within the neighborhood of *x* is given by *v*_*x*_(*v*_*x*_ *-1*)*/*2. The Cluster Coefficient (*C*_*x*_) for the node *x*, is defined as:

$$C_{x}=\frac{2*u}{v_{x}(v_{x}-1)}$$ (7)

The Average Cluster Coefficient, obtained by averaging the cluster coefficient of all the networks nodes, is a global metric often used to quantify the segregation at network level.

### Average shortest path length (PL)

Let *x* and *y* be two generic nodes of a network *V* of *n* nodes. Let *d*(*x*, *y*) be the shortest distance between the nodes *x* and *y*. We define the Average Path Length (*L*) as:

$$PL=\frac{2}{n(n-1)}\sum_{x\neq y}d(x,y)$$ (8)

This topological parameter is commonly used to evaluate the networks level of integration.

### Small world index (SWI)

To detect the emergence of small-world network [64], it is possible to combine the metrics previously introduced, defining the Small-World Index (SWI):

$$SWI=\frac{\frac{C_{net}}{C_{rnd}}}{\frac{L_{net}}{L_{rnd}}}$$ (9)

where *C*_*net*_ and *L*_*net*_ are the cluster coefficient and the path length of the investigated network, respectively, and *C*_*rnd*_ and *L*_*rnd*_ correspond to the cluster coefficient and the path length of random networks equivalent to the original network (i.e., with the same number of nodes and links). A SWI higher than 1 suggests the emergence of a small-world topology.

### Rich club

A graph representing a complex network is said to have a rich-club organization if the hub nodes of such a graph are more strongly connected with each other than expected by their high degree alone[42]. It is possible to infer such an organization by computing the Rich Club Coefficient (RCC).

The RCC value at a specific *k* level is computed by evaluating the cluster coefficient among the nodes with a degree higher than *k*:

$$RCC(k)=\frac{2E_{>k}}{N_{>k}(N_{>k}-1)}$$ (10)

where *N*_*>k*_ is the number of nodes with a degree higher than *k*, and *E*_*>k*_ represents the edges between them. Evaluating the RCC with *k* varying from 1 to the maximum degree allows to build the RCC curve. The RCC curve is normalized by the corresponding average value for a set of surrogated random neural networks equivalent to the investigated one (i.e., networks with the same number of nodes and edges). If the maximum RCC normalized coefficient value is higher than one, a privileged sub-network (i.e., a rich club) is found.

### Computational model

The network model was made up of 1000 neurons randomly connected. The dynamics of each neuron is described by the Izhikevich equations[65]. In the actual model, two families of neurons were taken into account: regular spiking and fast spiking neurons for excitatory and inhibitory populations, respectively (S2A Fig). The ratio of excitation and inhibition was set to 4:1 as experimentally founded in cortical cultures [8]. In the model, each excitatory neuron receives 100 connections from the other neurons (both excitatory and inhibitory) of the network. Such incoming connections reflect the same ratio of the neuronal population, i.e., 80% of excitatory and 20% of inhibitory links. (S2C Fig). Each inhibitory neuron receives 100 input only from excitatory neurons. Autapses are not allowed. All the inhibitory connections introduce a delay equal to 1 ms, otherwise excitatory ones range from 1 to 20 ms [66]. Synaptic weights were extracted from a Gaussian distribution with mean equal to 6 and -5 for excitatory and inhibitory weights (S2B Fig). Standard deviations were set to 1. Excitatory weights evolve following the spike timing dependent plasticity (STDP) rule with a time constant equal to 20 ms[67]. The spontaneous activity of the neuronal network was generated by stimulating a randomly chosen neuron at each time stamp injecting a current pulse extracted from a normal distribution (*I*_*stm*,*exc*_ = 11 ± 2; *I*_*stm*,*inh*_ = 7 ± 2). The network model was implemented in Matlab (The MathWorks, Natick, MA, USA), and each run simulates 1 hour of spontaneous activity.

### Cell culture, experimental set-up and experimental protocol

Cortical and striatal neurons were dissociated from rat embryos (E18) Sprague Dawley (Charles River Laboratories). The day before plating, Micro-Electrode Arrays (both MEA-4k and MEA-60) were coated with the adhesion proteins laminin and Poli-Lysine (Sigma-Aldrich). The final density of plating was about 1200 cells/mm^2^ for the MEA-60 and 700 cells/mm^2^ for the MEA-4k. MEAs were maintained for four weeks in a humidified incubator (5% CO_2_, 37°C) in Neurobasal medium supplemented with B27. More details about cell cultures can be found in previous works[50, 68]. Recordings were performed using two kinds of MEAs: (i) MEA-60 (Multi Channel Systems, Reutlingen, Germany) constituted by 60 planar Ti/TiN microelectrodes 200 *μ*m spaced with a diameter of 30 *μ*m and arranged in a 8 by 8 square grid (electrodes in the four corners are not present). (ii) MEA-4k (3Brain, Wadenswill, Switzerland) constituted by 4096 square microelectrodes 42 *μ*m spaced, 21 *μ*m side length, arranged in a 64 by 64 square grid. Recordings of spontaneous activity were performed during the fourth week *in vitro*. We recorded 1 hour of spontaneous activity at the sampling frequency of 10 kHz (MEA-60) and of 9046 Hz (MEA-4k).

### Data analysis

Data analysis was performed off-line using Matlab and C# (Microsoft, US). *Spike detection*. The algorithm used to detect extracellularly recorded spikes was the Precise Timing Spike Detection (PTSD) [69]. Practically, the detection was performed by setting three parameters: a differential threshold, evaluated as 8 times the standard deviation of the noise of each electrode; a peak life time period (set at 2 ms) and the refractory period (set at 2 ms). Spike sorting was not performed as it is often difficult to distinguish different shapes during bursts due to overlapping spikes [60]. *Burst detection*. Burst at single electrode level and network bursts were detected by using previously developed and validated algorithms. Single electrode bursting activity was detected by considering at least 5 spikes with a maximum Inter Spike Interval (ISI) of 100 ms [70]. *Functional connectivity and topological analysis*. The FNCCH used to infer functional connectivity, as well as the metrics used to characterize the topological features of the cortical networks (Small-World Index, Clustering Coefficient, average shortest Path Length) were collected in an update version of the SpiCoDyn software [71].

### Statistical analysis

Data are expressed as mean ± standard deviation of the mean. Statistical analysis was performed using Matlab. Since data do not follow a normal distribution (evaluated by the Kolmogorov-Smirnov normality test), we performed a non-parametric Kruskal-Wallis test. Significance levels were set at p < 0.001. In the box plot representation, the median value and 25^th^-75^th^ percentiles are indicated by the box, mean value is indicated by the small hollow square, and whiskers indicate 5^th^-95^th^ percentiles.

### Code availability

The developed FNCCH is available to the scientific community on the Neuroimaging Informatics Tools and Resources Clearinghouse, (NITRC) repository (http://www.nitrc.org/). In particular, the FNCCH has been embedded in a new release (v3.0) of the software tool SpiCoDyn (https://www.nitrc.org/projects/spicodyn/).

## Supporting information

S1 Data All the electrophysiological spike data recorded with the MEA-60 and MEA-4k devices and presented in the paper are available as binary files in HDF5 format in a compressed file. (RAR)

S1 Text Supplementary information include: S1. Post computation FNCCH filtering; S2. Computational Model; S3. FNCCH is able to identify topological properties of complex networks; S4. Identified inhibitory links depend on the recording time length; S5. FNCCH values are proportional to the strength of the connections; S6. Comparison with a Transfer Entropy based algorithm; S7. Spiking and bursting dynamics.
(DOCX)

S1 Fig FNCCH post filtering procedure. In this illustrative case, correspondent to weak correlation, the filtering procedure infers a negative value in the boundary region of the correlation window (black line) leading to a false positive inhibitory link. To avoid this, heuristic post filtering procedure is performed by a peak search re-applied in a smaller region of the correlation window (green line) discarding part of the tail. The resulting peak, in this example, is excitatory and with a shorter delay.
(TIF)

S2 Fig Computational model features and simulation results. **a,** electrophysiological patterns of excitatory (top) and inhibitory (bottom) neurons. **b,** Excitatory synaptic weights distribution at t = 0 (left side) and at the end of the simulation (right side). c, ach neuron receives (on average) 100 connections. In the case of excitatory neurons, the 80% of the incoming connections are excitatory, while the remaining 20% come from inhibitory neurons. **d,** Sketch of the permitted connections among the excitatory and inhibitory populations. **e,** MFR distributions. **f,** IBI distributions.
(TIF)

S3 Fig Functional connectivity estimation from a scale-free neural networks. **a**, Raster Plot and mean Instantaneous Firing Rate (IFR) representative of the simulated electrophysiological activity. **b,** Estimated functional in-degree distribution (red curve for excitatory links and blue curve for the inhibitory ones) and (inset) structural in-degree distribution of the implemented scale-free model. **c**, ROC functions for the inhibitory (blue curve) and the excitatory (red curve) links obtained by applying the FNCCH; the black curve, is related to only excitatory links extracted with the standard NCCH, is depicted for comparison. Corresponding AUCs are represented in the inset. **d**, MCC curves related to inhibitory and excitatory links computed by applying the FNCCH; the black curve, related to only excitatory links extracted with the standard NCCH, is depicted for comparison.
(TIF)

S4 Fig Percentage of the inhibitory links revealed by the FNCCH at the varying of the recording time length. (TIF)

S5 Fig Comparison of the FNCCH values extracted from the simulations of n = 10 *in silico* networks. The differences between true and false positive for both excitatory and inhibitory links are statistically different (p value<0.001, Kruskal-Wallis non parametric test).
(TIF)

S6 Fig DTE effective connectivity estimation relative to an in silico neuronal network. Functional links are estimated starting from the simulated multi-site electrophysiological activity. **a,** ROC curves relative to the total links (black), to the excitatory versus excitatory neurons’ links (red), to the inhibitory versus excitatory neurons’ links (blue) and to the to the inhibitory versus inhibitory (green). **b,** Correspondent AUCs. **c,** DTE weighted connectivity matrix.
(TIF)

S1 Table Topological parameters extracted from the *in silico* Scale Free neural network compared to a random one. (TIF)

S2 Table Spiking and bursting features of neuronal cultures coupled to MEA-4k. (TIF)
