# Supplementary material for: Identification of excitatory-inhibitory links and network topology in large-scale neuronal assemblies from multi-electrode recordings
Source: PLoS Comput Biol. 2018 Aug 27;14(8):e1006381. doi: 10.1371/journal.pcbi.1006381 (PMC6128636; doi:10.1371/journal.pcbi.1006381)
Supplement: S1 Text — Post computation FNCCH filtering; S2. Computational Model; S3. FNCCH is able to identify topological properties of complex networks; S4. Identified inhibitory links depend on the recording time length; S5. FNCCH values are proportional to the strength of the connections; S6. Comparison with a Transfer Entropy based algorithm; S7. Spiking and bursting dynamics. (DOCX) [file pcbi.1006381.s002.docx]

# Supplementary Information

Identification of excitatory-inhibitory links and network topology in large-scale neuronal assemblies from multi-electrode recordings

Vito Paolo Pastore^1^, Paolo Massobrio^1^, Aleksandar Godjoski^1,2^ and Sergio Martinoia^1,3^

^1^University of Genova, Dept. of Informatics, Bioengineering, Robotics and System Engineering, Via Opera Pia 13, 16145 Genova, Italy

^2^3Brain gmbh, Einsiedlerstrasse 32, 8820 Wädenswil, Switzerland

^3^CNR - Institute of Biophysics, Via De Marini, 6, 16149 Genova, Italy

**S1. Post computation FNCCH filtering**

The temporal occurrence of the extracted peak of the Normalized CCH (NCCH) represents the time delay of the identified connection between two neurons. When we deal with experimental recordings, the NCCH could be very jagged (Figs. 6f and 6g of the main text), with a shape characterized by oscillations in the central region of the correlation window and a typical decrease of the NCCH values in the borders (Fig. S1). The procedure we described in the Results section of the main text (Eq. 1), permits to extract the peaks evaluating their sign, but it is sensitive to a decrease in synchrony due to uncorrelated activity. Such a decrease appears at the boundaries of the correlation window (Fig. S1, black curves), and can be exchanged for a decrease in synchrony related to an inhibitory connection. Thus, we expect that in specific cases characterized by a very jagged correlogram (e.g., low firing rate), this procedure will introduce some mistakes in the inhibitory connections detection. We defined this artifact as tail effect on the FNCCH. For this reason, we implemented a post FNCCH filtering operation that allows us to account for the presence of these artifacts removing them from the set of identified inhibitory connections. More specifically, the filtering procedure consists in a few steps applied to every negative FNCCH values falling in one of the two boundary regions of the correlation window (defined as the 15% of half the amplitude of the correlation window, Fig. S1 black curves).

If such inhibitory detected link is an artifact due to the tail effect, the FNCCH values in the boundary region will be all negative and, likely, with a decreasing trend. In this case, the corresponding putative peak is discarded and a new search starts for another peak excluding the boundary regions. We then search for a peak in the central region of the correlation window that we called “peak re-computation window” (Fig. S1, green curve, by applying Eq. (1) (cf. Methods). Indeed, the “new” peak can be related either to an inhibitory effective connection or to an excitatory one. Conversely, if we find an increase in the FNCCH reaching a positive value before the end of the correlation window, we can state that the negative peak is correctly identified (i.e., an actual minimum).

**S2. Computational Model**

The model we developed to simulate *in vitro* network dynamics is made up of two neuronal populations whose electrophysiological patterns are modeled following the Izhikevich model[^1^](#_ENREF_1). In particular, excitatory neurons exhibit a regular spiking activity (Fig. S2a top), while inhibitory ones a fast spiking activity (Fig. S2a bottom).

These two families of neurons are randomly connected, but not all the interconnections are available: in particular, excitatory neurons can project to other excitatory and inhibitory neurons. Inhibitory neurons can hyperpolarize only excitatory neurons (Fig. S2d c). Finally, autapses are not allowed. Each excitatory neuron receives 100 connections from other neurons (both excitatory and inhibitory) of the network. Such incoming connections reflect the same ratio of the neuronal population, i.e., 80% of excitatory and 20% of inhibitory links. (Fig. S2c). Each inhibitory neuron receives 100 input only from excitatory neurons. The efficacy of the synaptic transmission is described by means of synaptic weights which are extracted from a Gaussian distribution with mean equal to 6 and -5 for excitatory and inhibitory weights respectively. Standard deviations have been set to 1. Excitatory weights evolve following a spike timing dependent plasticity (STDP)[^2^](#_ENREF_2) rule with a time constant equal to 20 ms^[2](#_ENREF_2)^ (Fig. S2b). The steady state value of the synaptic weight is reached after 5 minutes of the beginning of the simulation. The spontaneous activity of the neuronal network has been generated by stimulating a randomly chosen neuron at each time stamp injecting a current pulse extracted from a normal distribution (*I_stm,exc_* = 11 ± 2; *I_stm,inh_* = 7 ± 2). The network model has been implemented in Matlab (The Mathworks, Natik, US), and each run simulates 1 hour of spontaneous activity.

As reported in the main text, the simulated dynamics well mimics the spontaneous electrophysiological activity of mature *in vitro* cortical assemblies. Figs. S2e and S2f show the distributions of the mean firing rate (MFR) and inter-burst interval (IBI) of the excitatory and inhibitory populations.

**S3. FNCCH is able to identify topological properties of complex networks**

In the main text, we quantified the performances of the FNCCH by means of a computational model whose structural connectivity was ruled out by a random topology. To verify whether the developed connectivity method is able to recognize more complex network topologies, we simulated a neuronal network made of 1000 neurons connected by means of a scale-free topology and presenting at the same time small-world features. The percentage of excitatory and inhibitory neurons remain the same of the random network described in the main text (i.e., 80% excitation). No other features of the network (i.e., synaptic weights distribution, STDP time constants, background stimulation, etc.) have been changed. We simulated the spontaneous dynamics to verify whether we can correctly identify also this specific topology. Figure S3a shows a snapshot of the electrophysiological activity of the entire network. Compared to Fig. 1a, a more pronounced random spiking activity can be observed in accordance to what was previously shown[^3^](#_ENREF_3). The traces (red for excitatory and blue for inhibitory neurons) of the IFR display the presence of network bursting involving all the neurons. By looking at the degree distributions of both excitatory and inhibitory connections (Fig. S3b), we can qualitatively identify a power law distribution for both the sub-populations which resembles the original structural one (Fig. S3b inset). In addition, we found a value of SWI = 6.57 suggesting the emergence of small-world properties. Finally, Figure S6c and d show the performances of the FNCCH (compared with NCCH) to reconstruct the scale-free topology by means of the ROC curve[^4^](#_ENREF_4) (and its relative AUC) and MCC. The obtained results are a clear indication that FNCCH is able of correctly identifying the topology of the implemented network.

In Table S1, we summarize the results obtained with the topological analysis on one representative random and scale-free *in silico* network with small-world features. The FNCCH is able to reliably identify the topology for the two types of network models (scale free topology’s SWI >1, random topology’s SWI ≤1). Moreover, the scale free/small-world network appears more segregated (higher CC) than the random one, with no significant differences on the integration (PL values), as expected.

**S4. Identified inhibitory links depend on the recording time length**

We investigated the dependence of the detected and identified inhibitory links from the recording time length. In particular, we used the 10 *in silico* neural networks described in the main text, decreasing the recording time from 60 minutes to 10 minutes with a 10 minutes step. Fig. S4 shows the results. As we can see, at least 30 minutes of recording are necessary to estimate a percentage of functional inhibitory links close to the morphological one, set at 20%. This result is in good agreement with the experimental ones (Fig. 3f and Fig. 4f).

**S5. FNCCH values are proportional to the strength of the connections**

The Receiver Operating Characteristic (ROC) curve[^4^](#_ENREF_4) extracted from the *in silico* testing networks (Fig. 1c of the main text), shows that there is a striking difference between FNCCH values relative to true positives and false positives. This difference suggests that the hard threshold procedure can be considered a robust and valid method for discarding from the connectivity matrix, those values correspondent to spurious connections, and not to real connections. To prove the existence of a clear difference between false and true positives, we compared the mean values for both excitatory and inhibitory links (Fig. S5).

**S6. Comparison with a Transfer Entropy based algorithm**

In this section, we analyze the performances of the Transfer Entropy (TE) algorithm in relationship to its ability to detect and, eventually, to identify excitatory and inhibitory functional links. In particular, we chose the Delayed Transfer Entropy (DTE)[^5^](#_ENREF_5). Fig. S6a shows the ROC curve and the correspondent AUC (Fig. S6b) when DTE is applied to an *in silico* random network made up of 1000 neurons (cf., Methods).

The ROC curve relative to the total number of links (black line) displays a shape similar to the one correspondent to the NCCH (black line of Fig. 1c, main text). However, if we analyze separately excitatory and inhibitory links, the results change dramatically. If we restrict the detection only to the excitatory links, the DTE ROC curve greatly improves (Fig. S6a), together with the AUC value (Fig. S6b). The ROC curve related to the inhibitory links (Fig. S6a) shows a good TPR value higher than 0.4 in correspondence of a FPR of 0.01. Finally, the ROC curve relative to the links between inhibitory neurons (green curve) shows that only false positives are detected, since in the model there are no links interneurons. Moreover, these false detected connections correspond to higher values with respect to all the other detected functional links (as confirmed by the connectivity matrix, represented with false color in Fig. S6c) with the consequence of worsening the total links detection. Thus, as reported in the main text, it is indeed possible to “detect” inhibitory links with a TE based method, but is not possible to “identify” (i.e., to distinguish) excitatory and inhibitory links.

**S7. Spiking and bursting dynamics**

Tab. S2 displays a general overview of the spiking and bursting features of the three neuronal networks coupled to MEA-4k.

**References**

1 Izhikevich, E. M. Simple model of spiking neurons. *IEEE Transactions on Neural Networks* **6**, 1569-1572 (2003).

2 Song, S., Miller, K. D. & Abbott, L. F. Competitive Hebbian learning through spike-timing-dependent synaptic plasticity. *Nature neuroscience* **3**, 919-926 (2000).

3 Massobrio, P., Pasquale, V. & Martinoia, S. Self-organized criticality in cortical assemblies occurs in concurrent scale-free and small-world networks. *Scientific reports* **5**, doi:10.1038/srep10578 (2015).

4 Fawcett, T. An introduction to ROC analysis. *Pattern Recognition Letters* **27**, 861-874 (2006).

5 Pastore, V. P., Godjoski, A., Martinoia, S. & Massobrio, P. SPICODYN: A Toolbox for the Analysis of Neuronal Network Dynamics and Connectivity from Multi-Site Spike Signal Recordings. *Neuroinformatics* **16**, 15-30, doi:10.1007/s12021-017-9343-z (2018).
