# Supplementary figures and images for: Identification of excitatory-inhibitory links and network topology in large-scale neuronal assemblies from multi-electrode recordings
Source: PLoS Comput Biol. 2018 Aug 27;14(8):e1006381. doi: 10.1371/journal.pcbi.1006381 (PMC6128636; doi:10.1371/journal.pcbi.1006381)

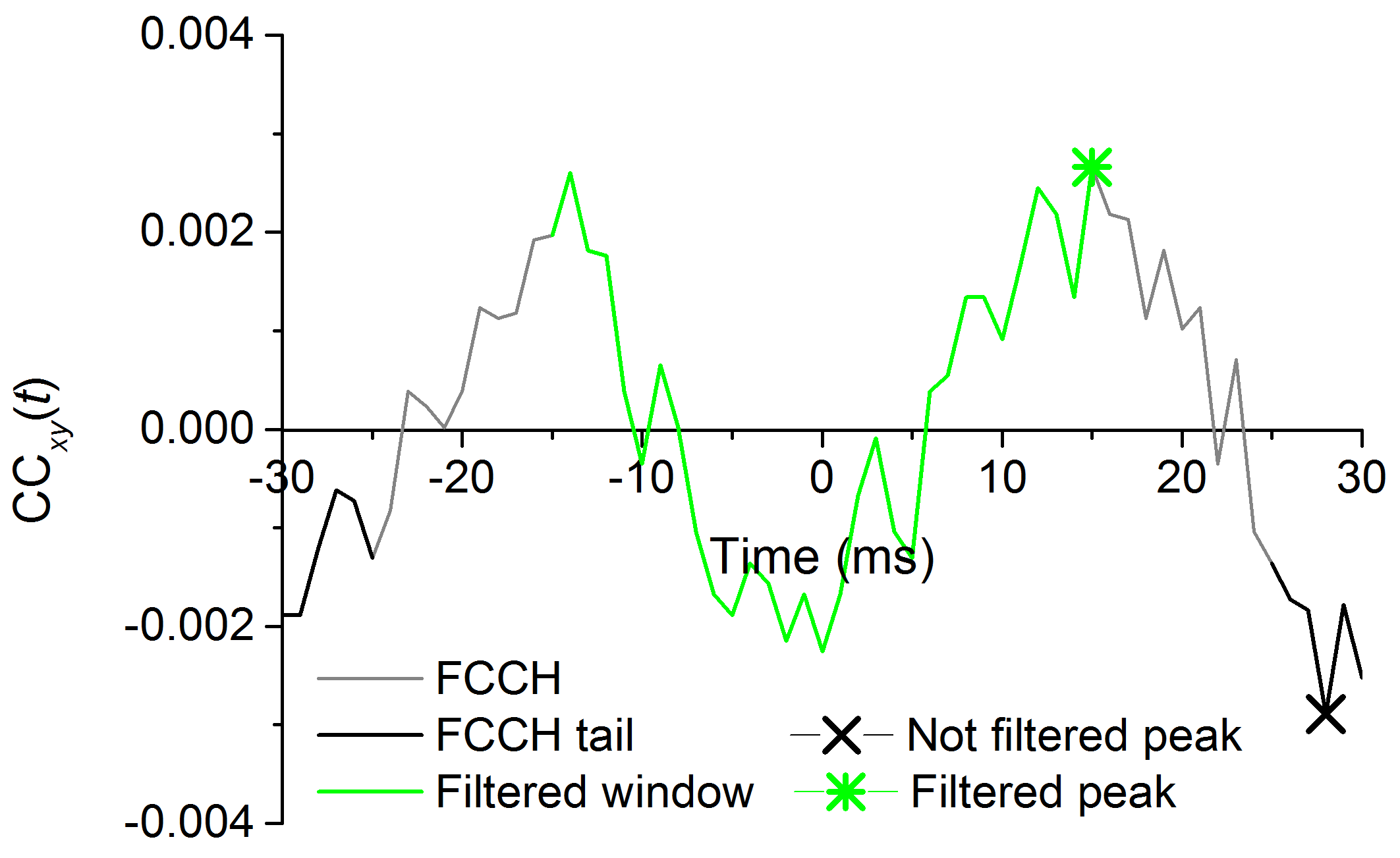

Supplement: S1 Fig — In this illustrative case, correspondent to weak correlation, the filtering procedure infers a negative value in the boundary region of the correlation window (black line) leading to a false positive inhibitory link. To avoid this, heuristic post filtering procedure is performed by a peak search re-applied in a smaller region of the correlation window (green line) discarding part of the tail. The resulting peak, in this example, is excitatory and with a shorter delay. (TIF) [file pcbi.1006381.s003.tif]

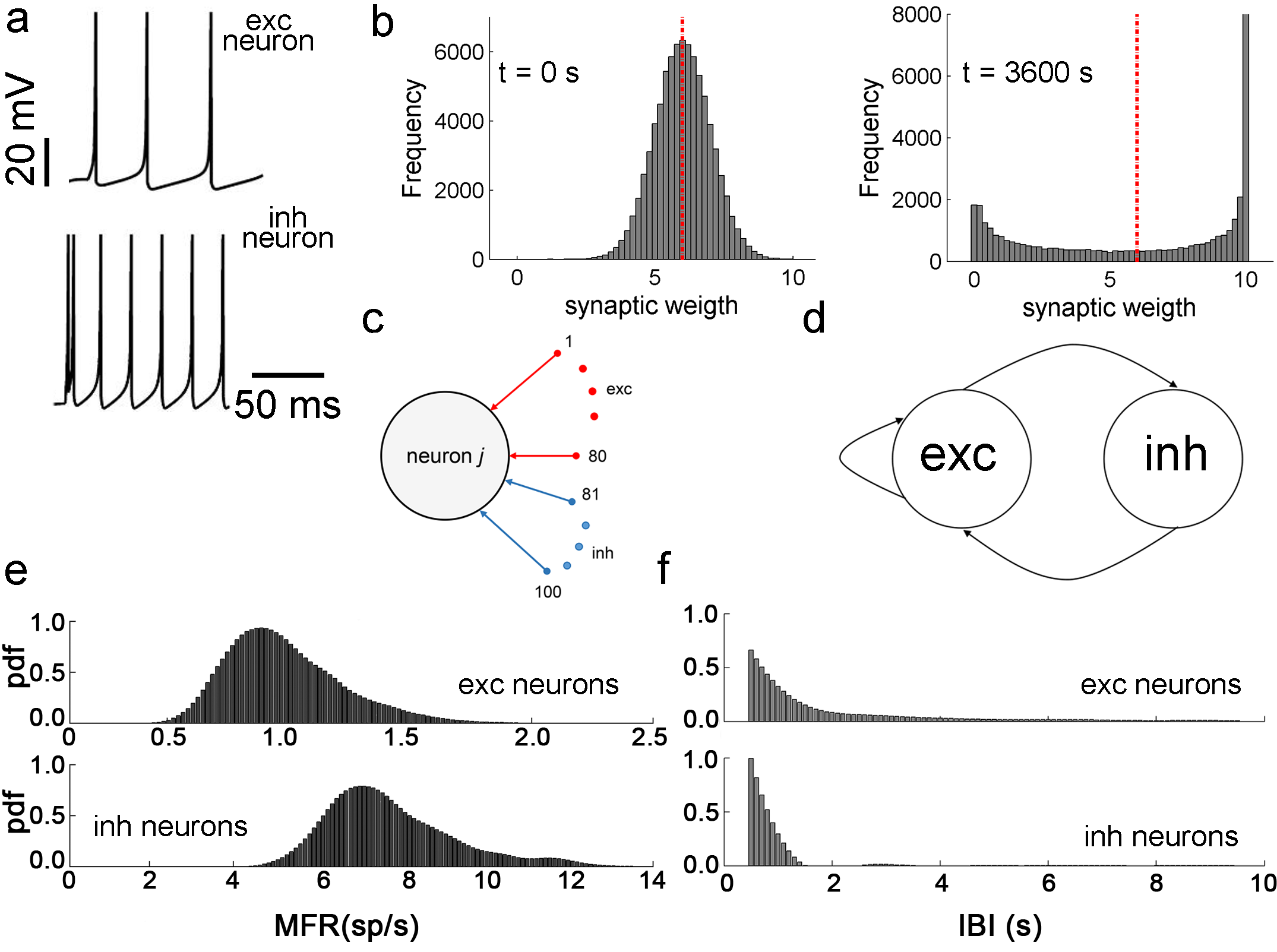

Supplement: S2 Fig — a, electrophysiological patterns of excitatory (top) and inhibitory (bottom) neurons. b, Excitatory synaptic weights distribution at t = 0 (left side) and at the end of the simulation (right side). c, ach neuron receives (on average) 100 connections. In the case of excitatory neurons, the 80% of the incoming connections are excitatory, while the remaining 20% come from inhibitory neurons. d, Sketch of the permitted connections among the excitatory and inhibitory populations. e, MFR distributions. f, IBI distributions. (TIF) [file pcbi.1006381.s004.tif]

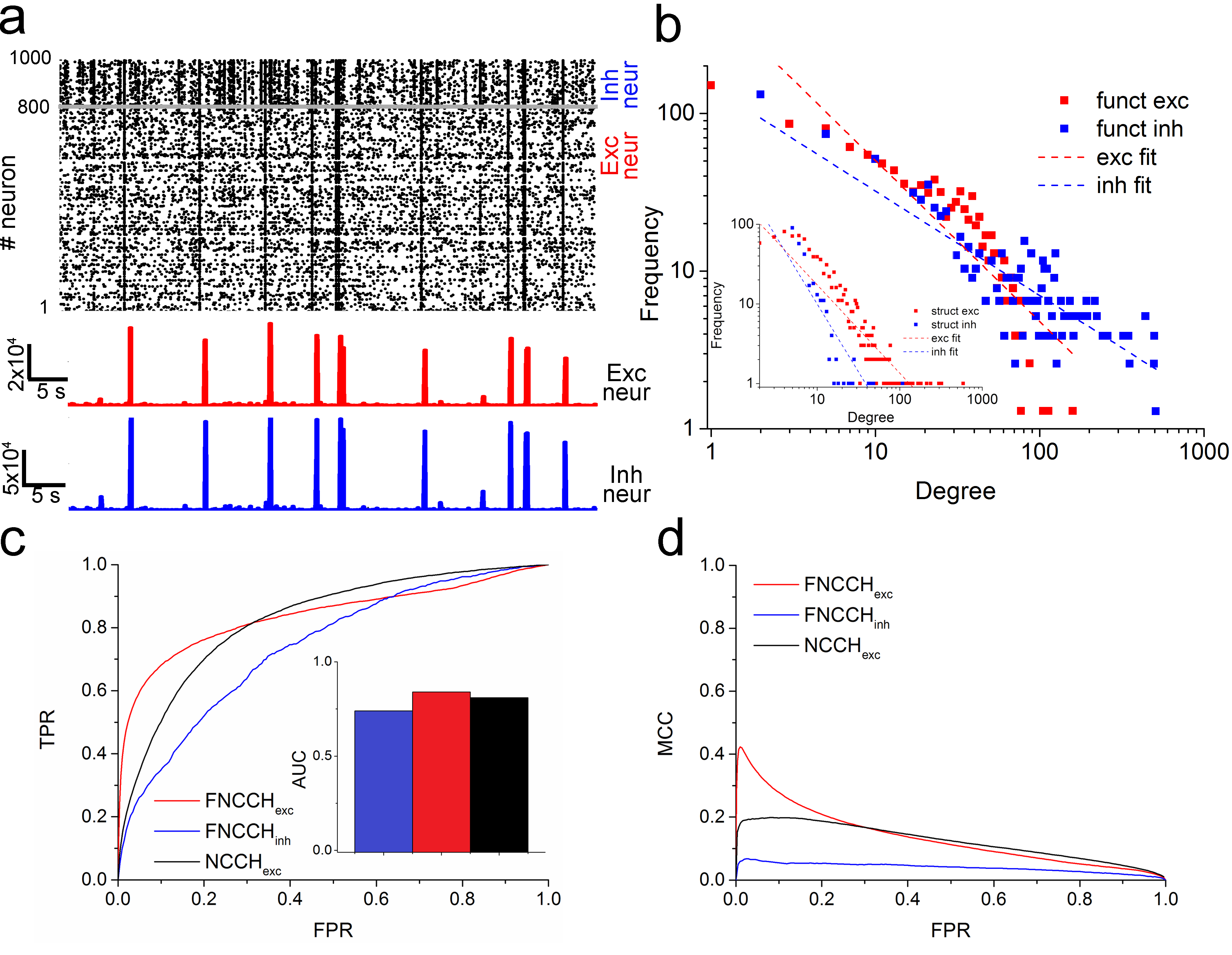

Supplement: S3 Fig — a, Raster Plot and mean Instantaneous Firing Rate (IFR) representative of the simulated electrophysiological activity. b, Estimated functional in-degree distribution (red curve for excitatory links and blue curve for the inhibitory ones) and (inset) structural in-degree distribution of the implemented scale-free model. c, ROC functions for the inhibitory (blue curve) and the excitatory (red curve) links obtained by applying the FNCCH; the black curve, is related to only excitatory links extracted with the standard NCCH, is depicted for comparison. Corresponding AUCs are represented in the inset. d, MCC curves related to inhibitory and excitatory links computed by applying the FNCCH; the black curve, related to only excitatory links extracted with the standard NCCH, is depicted for comparison. (TIF) [file pcbi.1006381.s005.tif]

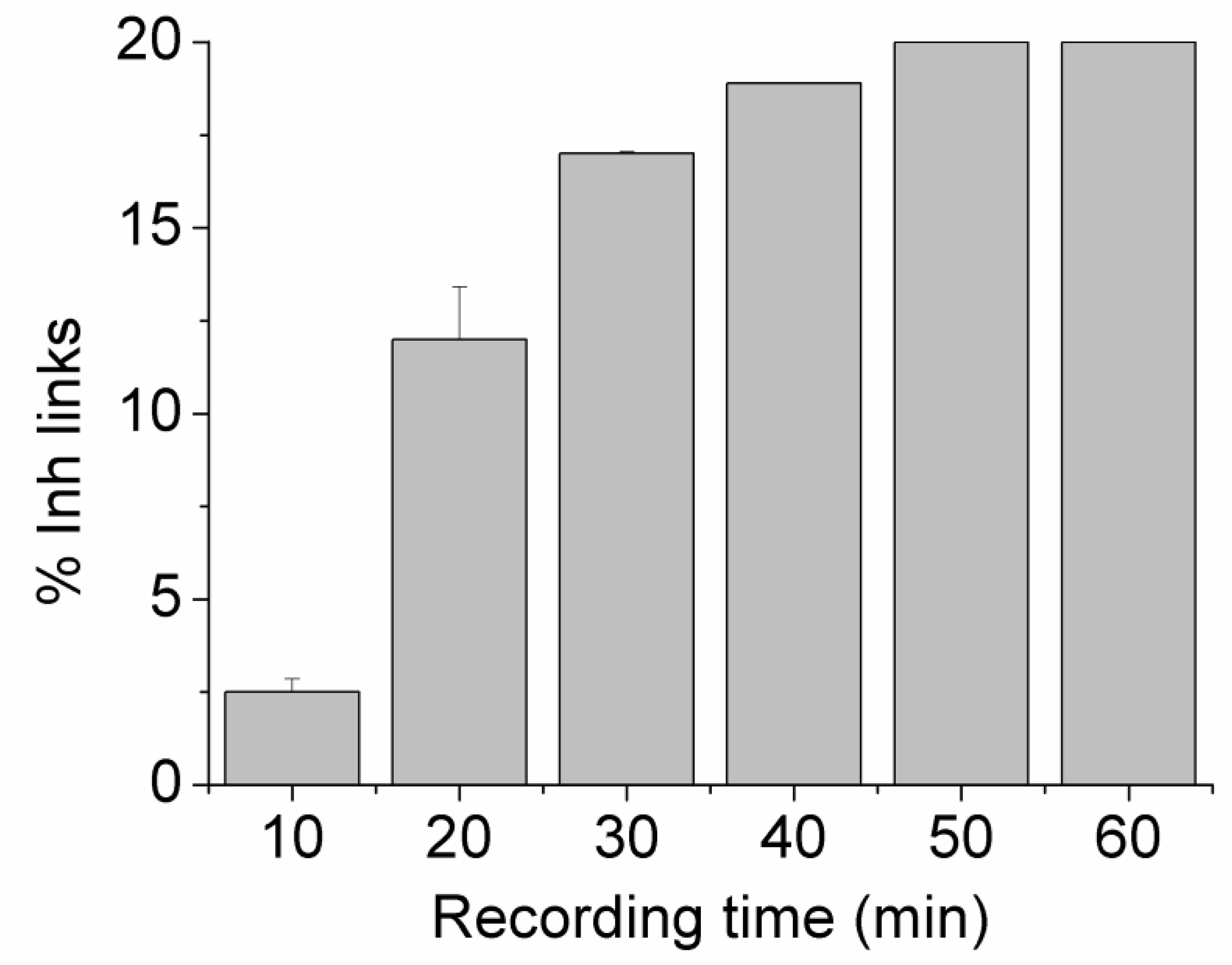

Supplement: S4 Fig — (TIF) [file pcbi.1006381.s006.tif]

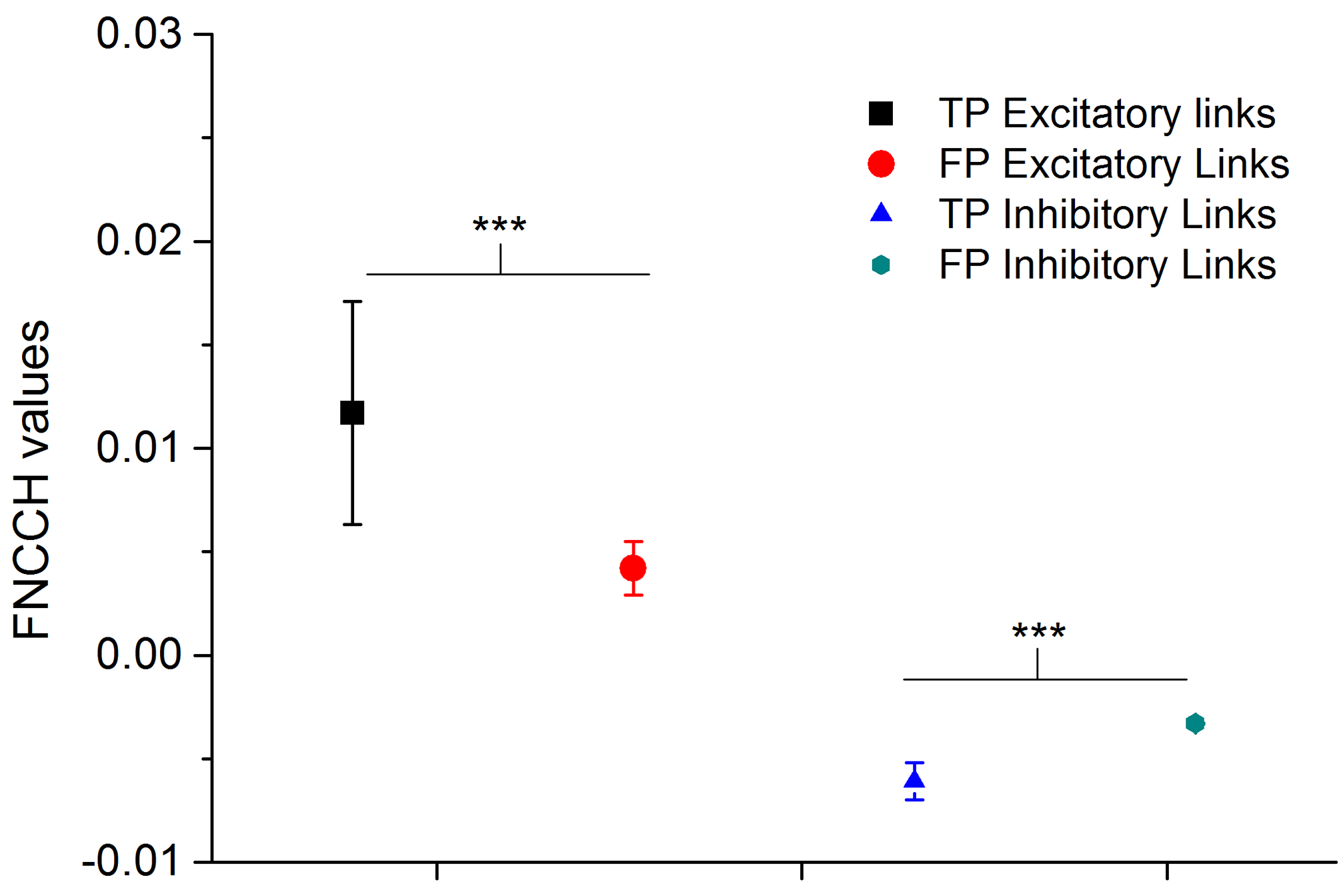

Supplement: S5 Fig — The differences between true and false positive for both excitatory and inhibitory links are statistically different (p value<0.001, Kruskal-Wallis non parametric test). (TIF) [file pcbi.1006381.s007.tif]

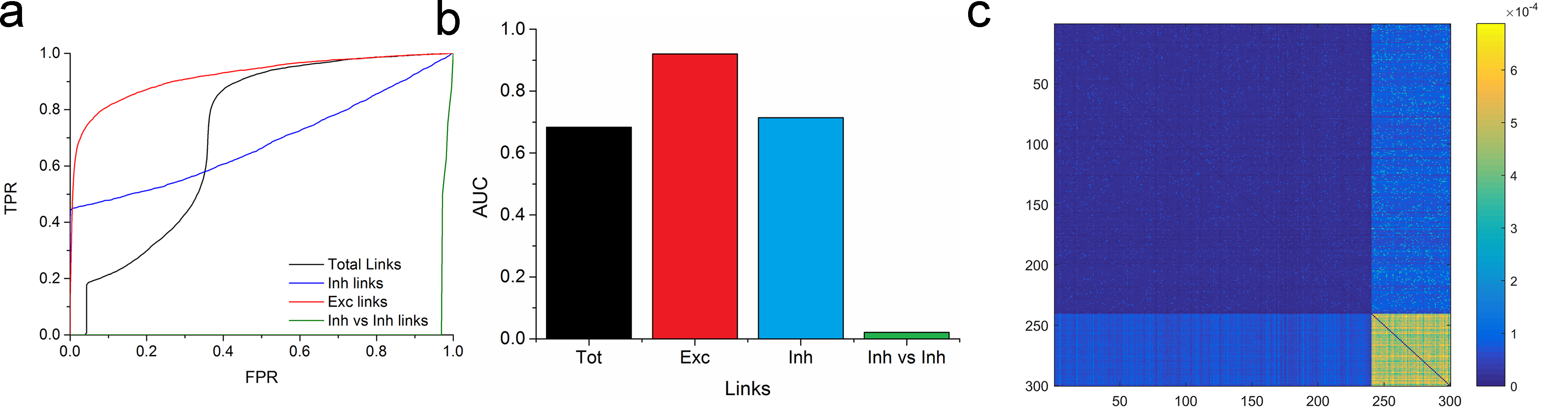

Supplement: S6 Fig — Functional links are estimated starting from the simulated multi-site electrophysiological activity. a, ROC curves relative to the total links (black), to the excitatory versus excitatory neurons’ links (red), to the inhibitory versus excitatory neurons’ links (blue) and to the to the inhibitory versus inhibitory (green). b, Correspondent AUCs. c, DTE weighted connectivity matrix. (TIF) [file pcbi.1006381.s008.tif]

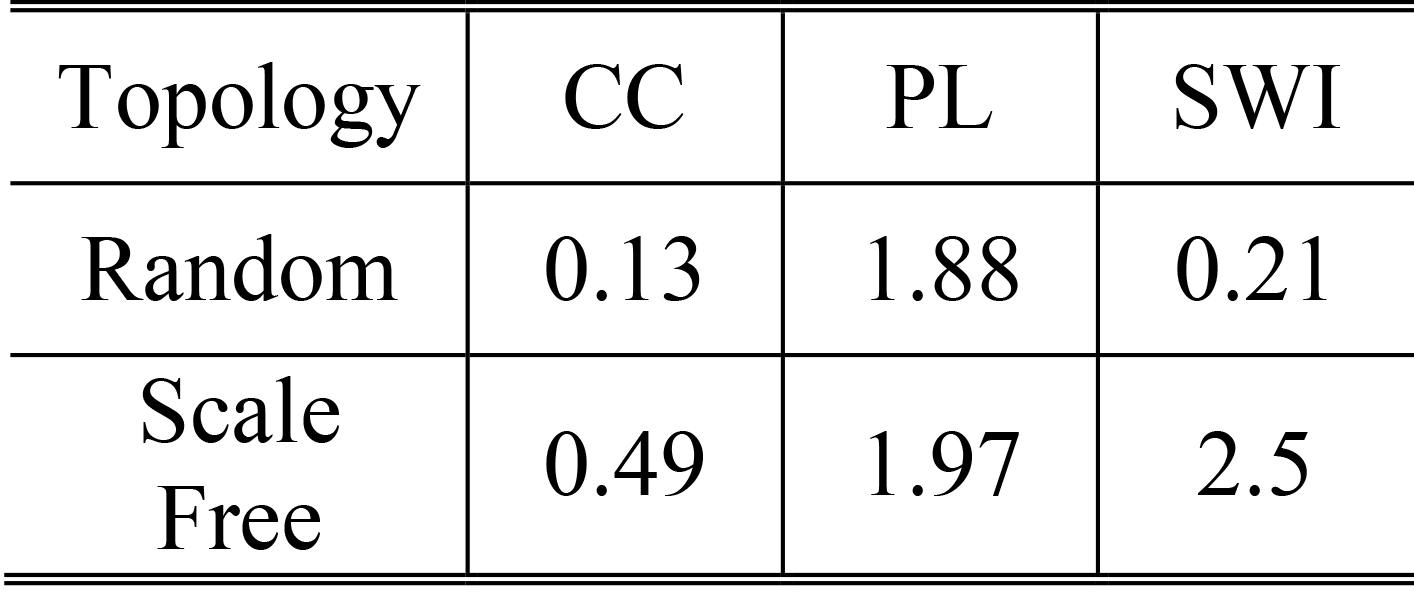

Supplement: S1 Table — (TIF) [file pcbi.1006381.s009.tif]

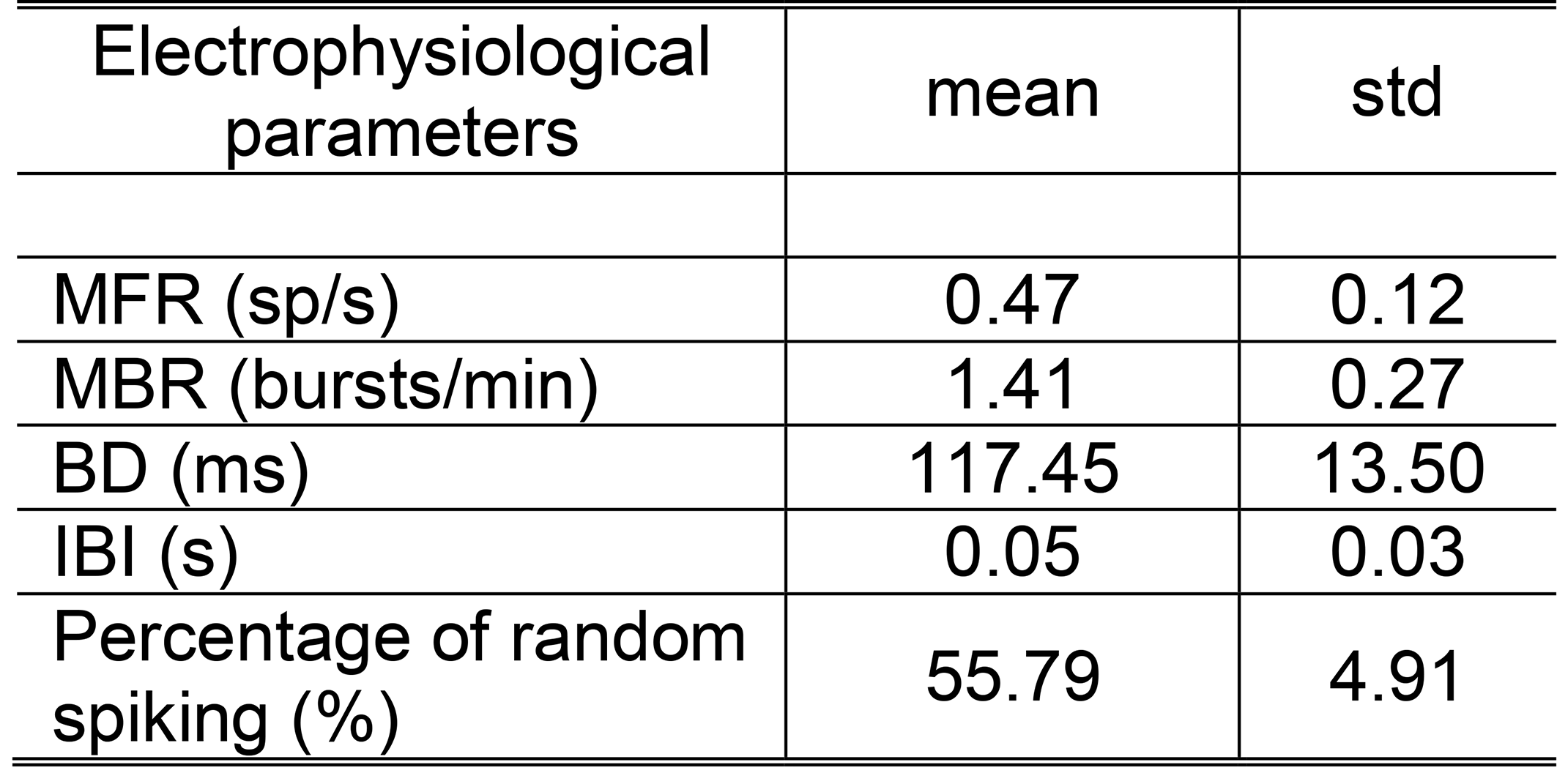

Supplement: S2 Table — (TIF) [file pcbi.1006381.s010.tif]
